# The effects of recycling pharmaceutical formulations in laser powder bed fusion 3D printing - the influence of physical phenomena on printing performance

**DOI:** 10.1016/j.ijpx.2025.100383

**Published:** 2025-08-30

**Authors:** Wessel Kooijman, Valerie R. Levine, Robbert J. Kok, Jonas Lindh, Julian Quodbach

**Affiliations:** a Division of Pharmaceutics, Utrecht Institute for Pharmaceutical Sciences (UIPS), Utrecht University, Universiteitsweg, 99 3584, CG, Utrecht, the Netherlands; b Division of Nanotechnology and Functional Materials, Department of Material Science and Engineering, Uppsala University, Uppsala SE-751 03, Box 35, Sweden

**Keywords:** Laser powder bed fusion, Selective laser sintering, Powder ageing, Powder recycling, Personalized medicine, Solid oral dosage forms

## Abstract

Laser powder bed fusion is an attractive technology for 3D printing objects in a powder bed and has been explored for printing pharmaceutical dosage forms, such as tablets. Recycling of non-sintered residual powder is not well understood, but is critical to prevent economic losses and improve the sustainability of this technique. We investigated the recyclability of three pharmaceutical formulations in the context of laser powder bed fusion of tablets. Three formulations consisting of common pharmaceutical polymers and the model drug indomethacin have been investigated up to ten print cycles. For each print cycle, powder and tablet samples were collected and analyzed for ageing phenomena. Results showed that polyvinyl alcohol and methacrylic acid-ethyl acrylate copolymer-based formulations were recyclable without changes in critical quality attributes of printed dosage forms for 5 and 9 cycles, respectively. A copovidone-based formulation showed a gradual increase in particle size over 10 cycles, resulting in a gradual decrease in tablet weight. This formulation was considered non-recyclable under the conditions evaluated in this work. The observed ageing phenomena were mainly related to changes in particle size, powder cohesion, and glass transition temperature. It is shown that considering powder ageing during formulation development is critical for optimal print performance and further development of laser powder bed fusion for pharmaceutical applications.

## Highlights

• Residual powder produced with selective laser sintering can be recycled.
• Powder ageing affects tablet quality and print consistency in SLS printing.
• Ageing of pharmaceutical formulations depends on material and process parameters.
• Changes in water content and particle size are major contributors to powder ageing.

## Graphical abstract

**Figure:**
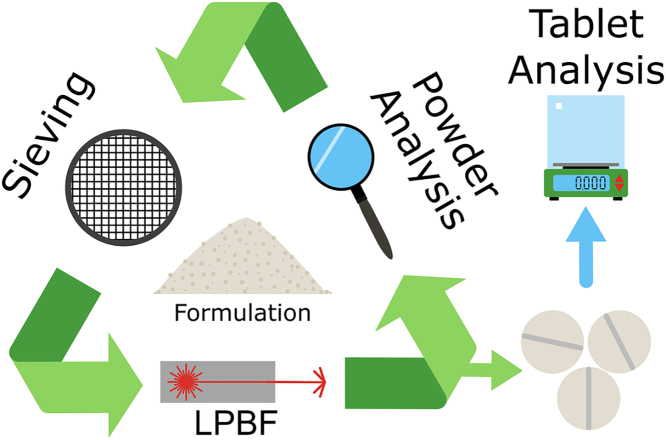

## 1 Introduction

In healthcare, multiple developments demonstrate the need for more personalized treatment of patients. For example, *Castro-Balado* et al. published a comprehensive review of the dosing of different antimicrobials in obese patients (Castro-Balado et al., 2024). Obese patients undergo pathophysiological changes that lead to changes in pharmacokinetics and pharmacodynamics. These changes can lead to under or over-exposure if standard dosages are used, which can either result in suboptimal therapeutic efficacy (in case of underdosing) or unwanted side effects or toxicity (in case of overdosing). Another example is given by *Swen* et al.*,* who showed that genotyping patients before treatment can justify dose adjustments of either the medication type and or the optimal dose in up to 50 % of the cases for specific medications (Swen et al., 2023). This pharmacogenomics-guided medication prescription can potentially lead to a 30 % reduction in clinically relevant adverse drug reactions, showing that personalized dosing in selected patients can be of great clinical relevance.

The previous examples show a clear need for dosage variation of common prescription drugs, which is currently not offered by industrial pharmaceutical manufacturing. Novel manufacturing technologies may address this challenge of personalized solid oral dosage form manufacturing. Currently, industrial compounding mainly relies on tablet presses, which can produce massive quantities of tablets per batch, but they lack the flexibility of easily adjusting the dosage. Pharmacy compounding is a very flexible process that can be tailored to the needs of individual patients. Common compounding strategies are the reformulation of dosage forms into liquid oral formulations or capsules with a personalized dose, but these are only feasible for relatively small batches. High-quality and safety standards and a lack of qualified personnel limit the relevance of small-scale pharmacy compounding in a systems approach to individualized dosing of common prescription drugs.

Additive manufacturing, or more commonly known as 3D printing, is a novel technology that is widely explored to produce personalized dosage forms. There are different 3D printing techniques investigated for the personalization of dosage forms, like fused deposition modelling (Abdelhamid et al., 2024; Kocabas et al., 2024), semisolid extrusion (Lafeber et al., 2021; Levine et al., 2024; Seoane-Viaño et al., 2021), binder jetting (Chang et al., 2020; Tan et al., 2023) and laser powder bed fusion (LPBF), also known as selective laser sintering (Tikhomirov et al., 2023a; Trenfield et al., 2018). LPBF has promising capabilities in the context of industrial-scale manufacturing of personalized and mass customized dosage forms.

LPBF printing has several advantages over other 3D printing techniques. LPBF printing allows for large print batches of 1000 tablets in smaller printers (10x10x10 cm) to easily fitting >50,000 tablets/batch for larger printers. The LPBF process lends itself well for large-scale production as the underlying processes of powder flow (Shah et al., 2023) and powder melting (Patil et al., 2024) are well understood for pharmaceutical formulations and powders. In addition, equipment is already on the market and used in healthcare for the manufacturing of prosthetics and medical devices (Ciocca et al., 2011; Di Giacomo et al., 2014; Morrison et al., 2015; Pattanayak et al., 2011; Sannomiya et al., 2008). Existing LPBF printers could be readily developed further to fulfill GMP requirements to safely print dosage forms at scale.

In contrast to LPBF, conventional pharmaceutical manufacturing techniques are optimized for mass production of fixed-dose formulations and offer limited flexibility for dose personalization. While upstream processes like granulation (Vadaga et al., 2024) or hot-melt extrusion (Li et al., 2024) can enhance powder properties or drug solubility, they still rely on downstream tablet pressing for dosage form production. This workflow requires specialized tooling, extensive development times, and batch-based quality control procedures (Leane et al., 2018). LPBF overcomes these constraints by enabling digital, on-demand production with no need for mechanical tooling changes or formulation adjustments, making it particularly suited for both mass customization and individualized therapy.

LPBF has only recently been explored as a method for the manufacturing of personalized dosage forms (Fina et al., 2018; Fina et al., 2017; Trenfield et al., 2018). LPBF printing is a powder bed fusion printing technique that utilizes a laser to fuse powder particles into a solid object. Powder layers are spread by a powder recoater, after which a laser is used to selectively fuse, i.e., sinter the powder particles together according to the object design. When the laser melts the powder, the liquified material flows into the contact zone between two particles, which is thermodynamically favorable due to the reduction in surface energy. The resulting connections can become strong, glassy connections upon the evaporation of moisture or the lowering of the temperature (Fitzpatrick et al., 2010; Haider et al., 2018). The process usually takes place at elevated temperatures to reduce the necessary energy input by the laser to sinter or melt the material, as well as to reduce unwanted phenomena like warping and balling (Guo et al., 2024). After the printing process has finished and the powder bed has cooled down to room temperature, the printed tablets can be retrieved by brushing away the residual material.

Due to the nature of the LPBF printing process, a large quantity of unsintered powder i.e., residual powder is produced, besides the printed tablets (de Alves et al., 2025; Machotová et al., 2024; Vendittoli et al., 2024; Wu et al., 2023). Proper recycling of this residual powder is essential to prevent economic losses and improve the sustainability of this technique (Alamos et al., 2020; Patel et al., 2021; Wu et al., 2023). The recycling of this powder is not trivial, however, as the material may be altered (i.e., it ages) when it is kept at the elevated temperatures necessary for printing. Ageing is a collective term for a variety of phenomena, e.g., thermal degradation, change in particle size and shape, enthalpy relaxation of the polymer chains, an increase in molecular weight due to post-condensation reactions and crosslinking leading to a higher melt viscosity, and a decrease in chain length due to chain cleavage leading to a reduction in viscosity. These changes in powder properties can lead to print failures when the aged powder is recycled (de Alves et al., 2025; Guo et al., 2023). Ageing of Polyamide 12 (PA12), an amide polymer powder commonly used in the industrial application of LPBF printing, has been extensively researched (Chen et al., 2018; Yang et al., 2023; Yang et al., 2020). The primary ageing phenomena for PA12 are thermal oxidation and post-condensation reactions. Ageing of PA12 is remedied by “refreshing” the aged powder by blending in fresh material. Aged material is commonly blended with 30–70 % fresh material (de Alves et al., 2025; Sanders et al., 2024).

In this work, an ageing study is performed on three pharmaceutical formulations suitable for LPBF printing. The formulations are based on pharmaceutical polymeric excipients that have been used as binders in LPBF studies and are structurally different from each other (Barakh Ali et al., 2019; Fina et al., 2017; Tikhomirov et al., 2023a). The materials are aged by recycling through multiple printing cycles, creating a realistic view of how formulations evolve during continuous printing, rather than relying on artificial ageing. During every printing cycle, samples of the residual powder and printed tablets were taken for analysis. The powder samples were analyzed for changes in thermal behavior, powder flow, particle size distribution, chemical structure and crystallinity. The tablets were analyzed for changes in weight, size, and breaking force. In addition, the robustness of our findings on the ageing of pharmaceutical powders is challenged by performing the ageing study with two different printers: one equipped with a CO₂ laser and the other with a blue diode laser.

## 2 Materials and methods

### 2.1 Materials

The powder blends (Table 1) used in this study are comprised of the following polymers: polyvinyl alcohol (PVA) (Parteck MXP 4–88, Merck, Germany), copovidone (PVPVA) (Plasdone S-630 Ultra, Ashland, Germany), and Methacrylic acid-ethyl acrylate copolymer (MAEA) (Eudragit L100–55, Evonik, Germany). Fumed silica (Aerosil 200, Evonik) was used as a glidant and a red colorant (Candurin® NXT Ruby Red, Merck) as an energy absorber for the diode laser. To ensure comparability between printers with different laser systems, the colorant was added to all formulations. The non-steroidal anti-inflammatory drug indomethacin was used as an active pharmaceutical ingredient (API, TCI, Belgium). 1.5 to 2 kg of the powder blends (Table 1) were prepared, the components were weighed separately and the polymers were subsequently sieved through a sieve (600 μm) to remove agglomerates. The materials were mixed in two steps in a Turbula mixer (Turbula T2F shaker) (WAB, Switzerland). In the first step, the polymer, indomethacin and pigment are mixed for 10 min at 72 rpm. In the second step, the SiO_2_ was blended in for 10 min at 49 rpm and the material was subsequently sieved (315 μm) to remove potential agglomerates again.

**Table 1:** Formulation percentages for the different materials. * For the PVA blend used in the SnowWhite^2^,0.56 % was used.

Material	Amount (%)
Indomethacin	10
Polymer	88.5
SiO_2_	0.5 / 0.56^⁎^
Pigment	1

### 2.2 Tablet design and printing pattern

For printing, a Kit SLS 3D printer (Sintratec AG, Switzerland) and a Sharebot SnowWhite^2^ SLS 3D printer (Sharebot, Italy) were used. The powder reservoirs of the printers were loaded to full capacity (i.e. with 1.5–2 kg for both printers) after which cylindrical tablets d = 10 mm, and h = 4 mm were printed*.* Tablet shapes were designed in AutoCAD® 2024 (Autodesk, USA) software, while slicing of the 3D objects into layers of 125 μm on the z-axis was done using Cura 5.2.1 (Ultimaker, The Netherlands). The tablets were laid out in a checkerboard pattern with in total 32 or 18 tablets spanning a square surface of 8 × 8 cm or 7 × 7 cm respectively. The 32-tablet pattern can be observed in Fig. 3B.

### 2.3 Printing and ageing process and sampling

After the tablets were printed in the first layers, the printer kept spreading and heating new layers without sintering the powder bed with the laser. This was done until the powder reservoirs were empty and all the powder had undergone a spreading and heating cycle. After each printing cycle, tablets were removed for analysis. In addition, a powder sample of 100 ml was taken after cycles 1, 3, 5 and 10 while a 50 ml sample was taken after cycles 2, 4, 6, 7, 8 and 9. To ensure a sufficient supply of powder until cycle 10, it was not possible to take a 100 ml sample for each cycle. After the samples were taken, the remaining powder was carefully passed through a 315 μm sieve in each cycle using a playing card to ensure consistency. In the case of the PVPVA formulation, a cake formed that was manually broken apart with minimal force prior to sieving, demonstrating that the cohesion between particles was weak and reversible. For each sieving step, a negligible amount of material (< 0.1 % weight) was discarded. After sieving, the materials were loaded into the printer for the subsequent printing cycle.

The material was recycled and printed until one of two conditions was met: 1) the material successfully underwent 10 printing cycles or 2) the material aged to the point that printing failed before reaching cycle 10. After one of these conditions was met the leftover material was blended with fresh powder in 1:1 ratio to investigate if printability could be restored for one last print cycle. The complete cycling experiments were done once for each material. Mean values and standard deviations are calculated based on the analysis of multiple tablets and powder samples from the same print batch.

A second printing trial was performed where 800 g of PVA and MAEA formulations were prepared replacing the indomethacin with more of the same thermoplastic polymer. The formulations were cycled for three cycles each and 50 ml powder samples were taken before each printing cycle.

For clarity, cycle 0 refers to the use of fresh (virgin) powder, whereas cycle 1 and higher refer to the powder and tablets recovered after the corresponding number of print cycles (e.g., cycle 1 refers to material recovered after the first print cycle). This naming convention is used consistently throughout the manuscript.

### 2.4 Printing parameters

The employed polymers have different thermal characteristics, and the print settings were optimized separately for each formulation. The printers used in this study also operate with different laser systems. The SnowWhite^2^ (Sharebot, Italy) uses a 14 W CO_2_ laser while the Kit printer (Sintratec AG, Switzerland) uses a 2.3 W blue diode laser. Most pharmaceutical formulations readily absorb the infrared light emitted by the CO_2_ laser (λ = 10.64 μm). The blue light (λ = 445 nm), however, is hardly absorbed by white powders. A colorant was therefore added to all formulations to aid laser absorption, independent of the employed printer, to ensure comparability. The print settings of the two printers, consequently, differ for the same material. The print settings for the different formulations and printers are shown in Table 2.

**Table 2:** Print parameters used for each printer and material.

	Kit			Snowwhite^2^		
Polymer	PVA	MAEA	PVPVA	PVA	MAEA	PVPVA
Layer Height (μm)	125	125	125	125	125	125
Laser Scan Speed (mm/s)	200	200	200	480	2640	1320
Laser power (W)	2.3	2.3	2.3	2.1	4.2	4.2
Surface Temperature (°C)	125	130	112	115	110	100
Chamber Temperature (°C)	75	70	75	N/A	N/A	N/A

### 2.5 Characterization

#### 2.5.1 Particle size analysis and powder rheology

The particle size distribution was measured using a Mastersizer 3000 equipped with an aero S unit and the standard Venturi disperser. The feed rate, air pressure and hopper gap were adjusted to obtain obscuration rates between 0.5 and 8 % (Table 3). The settings for PVA deviate from the others to maintain suitable dispersion and measurement conditions. Background measurements were taken for 30 s after which the samples were measured for 10 s per sample.

**Table 3:** Mastersizer 3000 settings used for the analysis of the different formulations.

	PVA	PVPVA	MAEA
Feed rate (%)	35	40	40
Air pressure (barg)	0.5	1.0	1.0
Hopper gap (mm)	2.0	3.0	3.0

Powder rheology has been assessed with a Granudrum (Granutools, Belgium) for fresh powder, and powders retrieved after cycles 1 and 10. The sample cylinder with an inner diameter of 84 mm was filled with 52.5 ± 0.5 ml powder and subsequently rotated around its axis at an angular speed of 2, 5, 10, 14,18, 22, 26, 30, 45 and 60 rpm. At each angular velocity, a 5-megapixel CMOS camera took 40 images at 1 frame per second. The angular speed sequence is measured twice. Once accelerating and once decelerating through the sequence. Measurements were performed at 65 °C for the MAEA and PVPVA, and 60 °C for the PVA. Image analysis, as explained by Neveu et al., yields a cohesive index (CI) (Neveu et al., 2022).

#### 2.5.2 Solid state analysis

Water content was determined using thermogravimetric analysis (TGA). Samples of 10–15 mg of material were loaded into an open aluminum pan (100 μl) and the measurements were performed on a Mettler-Toledo TGA/DSC 3+ (Mettler, Switzerland) and TGA Q50 (TA instruments, USA). On the Mettler-Toledo TGA/DSC 3+, samples were heated from 25 °C to 200 °C at a heating rate of 10 °C/min under 200 ml/min N_2_ flow. On the TGA Q50, samples were heated to 80 °C at a heating rate of 10 °C/min after which the sample was kept at an isotherm for 20 min. Measurements were performed under 60 ml/min N_2_ flow. The different methods are not expected to lead to any significant difference in results and are therefore not distinguished in this work.

Differential scanning calorimetry (DSC) measurements were performed on a Discovery DSC (TA instruments). Samples of 4–8 mg were placed in 20 μl aluminum pans and crimp sealed with non-hermetic lids. The samples were heated at a heating rate of 10 °C/min from 25 to 80 °C to remove moisture without reaching the glass transition temperatures (T_g_) of the dry polymers, followed by cooling the temperature to 0 °C at a cooling rate of 10 °C/min. In a second heating ramp samples were heated from 0 °C to slightly below the degradation temperature (180–225 °C) at a heating rate of 10 °C/min. The measurements were performed under a nitrogen flow of 50.0 ml/min. The T_g_ and melting points are reported with their onset temperature.

Modulated differential scanning calorimetry (mDSC) measurements were performed on a Discovery DSC (TA instruments). Samples of 5–10 mg were placed in 20 μl aluminum pans and crimp sealed with hermetic lids. The samples were heated from 10 to 200 °C with a heating rate of 2 °C/min. The modulated temperature amplitude was 1.0000 °C and had a period of 40 s. The measurements were performed under a nitrogen flow of 50.0 ml/min. The reversing heat flow was used to determine the onset of the T_g_. The separation of reversing and non-reversing heat flow allows for a more accurate determination of the glass transition temperature.

X-ray powder diffraction (XRPD) patterns were recorded using a D2 PHASER (Bruker, USA) with a Lynxeye detector using Co K_α_ or Cu K_α_ radiation. Measurements were taken with 2Θ = 10–60° at 0.01 increments with 1 s per increment. The poly (methyl methacrylate) (PMMA) sample holder was spun around its axis at 15 rpm during the measurements.

#### 2.5.3 Fourier transform infrared spectroscopy

To study the formulations and potential degradation products, attenuated total reflectance (ATR) Fourier transform infrared (FTIR) spectra were recorded on a SpectrumTwo (PerkinElmer, USA). The sample size was 5–10 mg and measurements were taken from 450 to 4000 cm^−1^. The total range was scanned 16 times and the average was taken. Subsequently, an ATR correction and baseline correction were performed to yield the final transmission spectrum.

#### 2.5.4 Tablet analysis

Tablet height, diameter and breaking force were measured in accordance with Ph. Eur. 2.9.8 using a MT50 (Sotax, Switzerland) or PTB 311E 800 (Pharmatest, Germany). The tensile strength was then calculated with the formula shown below:

(1) $$T=\frac{2F}{\mathrm{\pi Dt}}$$

where F is the diametrical breaking force, D is the tablet diameter and t is the tablet thickness. (Fell and Newton, 1970).

Scanning electron microscopy (SEM) pictures were taken with a Phenom ProX (Thermo Fisher Scientific, USA) using an acceleration voltage of 10 kV and a back scattering detector. The uncoated samples were measured on a charge reduction sample holder.

### 2.6 Statistical analysis

Statistical relevance (*p*-values) of results was determined using a one-way ANOVA.

## 3 Results and discussion

The main manuscript focuses on data related to the SnowWhite^2^ printer, with supporting information section S1 providing additional details and complementary analyses for this dataset. Section 3.1 begins with a visual comparison of the tablets produced using SnowWhite^2^, after which the results are discussed separately for each formulation. For all formulations, FTIR and XRPD showed no changes in crystallinity or chemical degradation, and the corresponding data can be found in the Supporting Information (**Fig. S1–S3**).

Samples printed using the Sintratec Kit printer exhibited large and inconsistent variations both within and between print cycles, preventing any meaningful interpretation of the data. Details regarding the printing and analytical results obtained with the Kit printer are provided in Supporting Information **Sections S2 and S3**.

### 3.1 Sharebot SnowWhite2

**Fig. 1:**
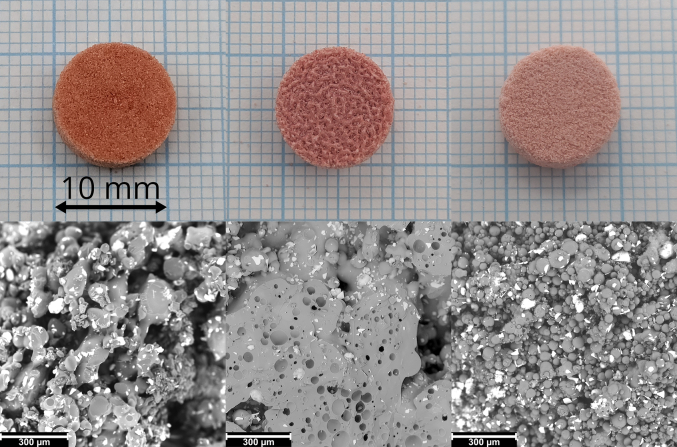
The top row shows pictures of the top of the tablets printed by SnowWhite^2^ from left to right PVA, PVPVA and MAEA. The bottom row shows SEM pictures of the cross-section of those tablets in the same material order, highlighting the morphology in greater detail.

**Fig. 2:**
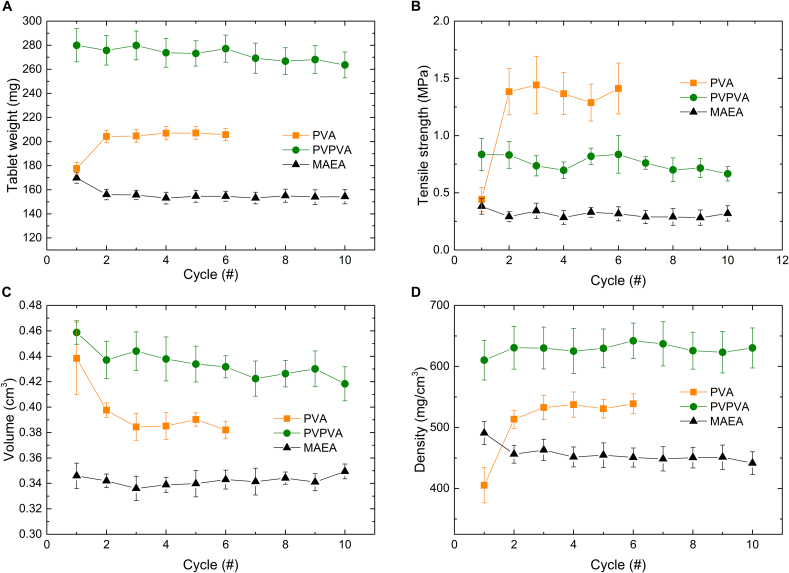
The average tablet weight **(A)**, tensile strength **(B)**, tablet volume **(C)** and tablet density **(D)** for the PVA, PVPVA and MAEA over the printing cycles printed in the SnowWhite^2^ printer. Values shown are based on a continuous measurement of the same starting powder; the mean ± standard deviation (s) are internal replicates (tablets from the same batch). The average tablet weight of PVA and MAEA was measured with n = 32, while PVPVA was measured with n = 18. For the tensile strength, volume and density, PVA and MAEA were measured on the MT50 with n = 10, while PVPVA was measured on the MT50 with n = 8.

The tablets printed on the SnowWhite^2^ from the three different formulations are shown in Fig. 1**, top**. The different tablet morphologies and colors highlight the different melting/sintering behavior of the polymers. The differences in melting and sintering behavior are visible in greater detail in the SEM images (Fig. 1**, bottom**) taken from the cross-section of the different tablets. The colorant can be recognized by its white, flaky appearance while the indomethacin particles are difficult to distinguish from the polymer. The PVA tablet exhibited visible thin solid bridges connecting the original polymer particles, with large pores throughout the structure. In contrast, the PVPVA tablet was fully fused into large solid sections, featuring macroscopic pores between them. Meanwhile, the MAEA tablet formed subtle solid bridges that are difficult to discern, accompanied by microscopic pores. Fig. 2 shows the tablet weight, volume density and tensile strength of the ageing experiment performed in the SnowWhite^2^ printer for the three formulations. The materials will be discussed in the same order as the images in Fig. 1 starting with PVA.

#### 3.1.1 Polyvinyl alcohol (PVA)

The PVA formulation could be successfully printed for six cycles (Fig. 2A, orange). The printing series showed a significant (*p* < 0.0001) difference in tablet weight between cycle 1 and 2. The tensile strength showed a similar change where there is an initial increase in tensile strength from printing cycle 1 to printing cycle 2 (p < 0.0001) after which the tablet tensile strength remained constant (Fig. 2B, orange).

**Fig. 3:**
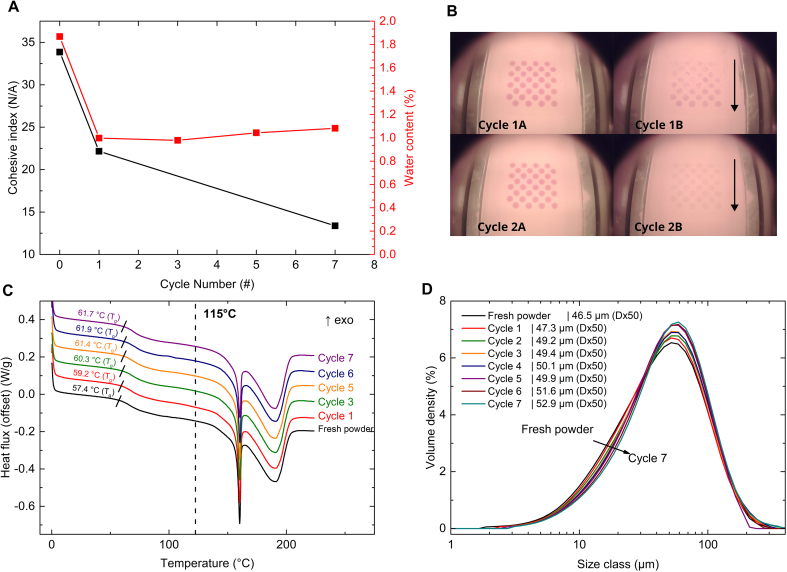
**A)** Cohesive index at 2 rpm (data extracted from **Fig. S4A)** and water content (data extracted from **Fig. S5A**) over the ageing cycles. The full datasets are provided in the corresponding supplementary information figures, while only the relevant portions are displayed here for clarity. **B)** The upper images show powder surfaces during the first printing cycle before (cycle 1 A) and after (cycle 1B) the deposition of a new powder layer. The lower images show the same for the second print cycle. In Cycle 1B, the previously molten layer is not uniformly covered, resulting in a spotted pattern on the surface. In Cycle 2B, the previous lasered layer is completely covered and a uniform layer is formed. **C)** Thermograms of the PVA formulation over the printing cycles measured after drying. The dashed line indicates the surface temperature during printing. The T_g_, defined as the onset of the transition, is indicated with a small black line. The seventh cycle corresponds to the failed print cycle. **D)** Particle size distributions of the PVA formulation over the print cycles. The seventh cycle corresponds to the failed print cycle

The initial increase in tablet weight can be attributed to a decrease in cohesive index from 34 for the fresh powder (cycle 0) to 22 for powder recycled once (cycle 1) (Fig. 3A, black). A cohesive index of 25 has been suggested as a threshold for proper layer spreading (Baesso et al., 2021; Understanding and Improving Powders Spreadability For a Recoater Process in Additive Manufacturing, 2020). The fresh powder exceeds this spreadability threshold and the cohesive index decreases below it after one printing cycle.

This decrease in cohesive index, i.e., increase in powder flow, matches the visual observation during printing. The fresh powder in cycle 1 forms a non-uniform powder bed upon spreading, whereas the recycled powder in cycle 2 results in a uniform and consistent layer (Fig. 3B). We assume that the improved powder flow leads to denser powder layers, resulting in denser, heavier, and stronger tablets. Fig. 3A shows a clear correlation between the water content and cohesive index. To gain more insight on the influence of the water content on the tablet weight and cohesive index, an additional cycling experiment was performed with the PVA formulation excluding indomethacin. This experiment was performed to see if the change in tablet weight from cycle 1 to cycle 2 is reproducible and to identify the influence of moisture on the T_g_ of the polymer. The results of this experiment are discussed in Section 3.1.3, as the MAEA formulation displayed an opposite but similar initial change in tablet weight.

After the initial increase, the tablet weight remains consistent over the cycles until cycle 7 where the material becomes unprintable due to dragging and warping issues. The cause for this remains elusive because there have been no considerable changes in the powder properties between cycle 6 and cycle 7. The T_g_ of the material steadily increased from 57.4 °C for fresh material to 61.9 °C after six print cycles (Fig. 3C). In addition, the particle size increased slightly from a Dx50 of 46.5 μm for fresh powder to 51.6 μm after six ageing cycles (Fig. 3D).

Subtle changes in polymer viscosity and melt rheology could reduce cavity formation or increase warping, ultimately leading to print failure (Tikhomirov et al., 2023a). The slight increases in particle size and T_g_ suggest that there are subtle changes in the physical and possibly chemical state of the powder particles. To further investigate these effects, melt rheology measurements and thermal imaging techniques could be employed in future research.

The PVA formulation performed consistently for five consecutive print cycles, which is considered sufficient because, in practice, material will never be fully recycled for more than 5 cycles and it will be continuously refreshed by blending with fresh powder.

#### 3.1.2 Copovidone (PVPVA)

The PVPVA formulation was successfully printed for 10 print cycles with an 18-tablet pattern. The 18-tablet pattern was printed because no print parameters were found where both the tablets on the in and outside of the 32-tablet pattern were printable. Tikhomirov et al. show that there is a temperature gradient in the build platform. (Tikhomirov et al., 2023b) It is hypothesized that there is a similar temperature gradient on the build platform of the SnowWhite^2^ printer, leading to the powder being colder on the outside and having a hotspot in the middle of the platform. This can lead to differences in tablet weight, as is shown for the 18-tablet pattern in **Fig. S6,** and in more severe cases, it can lead to tablet dragging and warping, as was observed for the 32-tablet pattern. The temperature gradient also led to variations in tablet weight for the PVA and MAEA formulations, but these variations did not result in printability issues for the 32 tablet pattern.

**Fig. 4:**
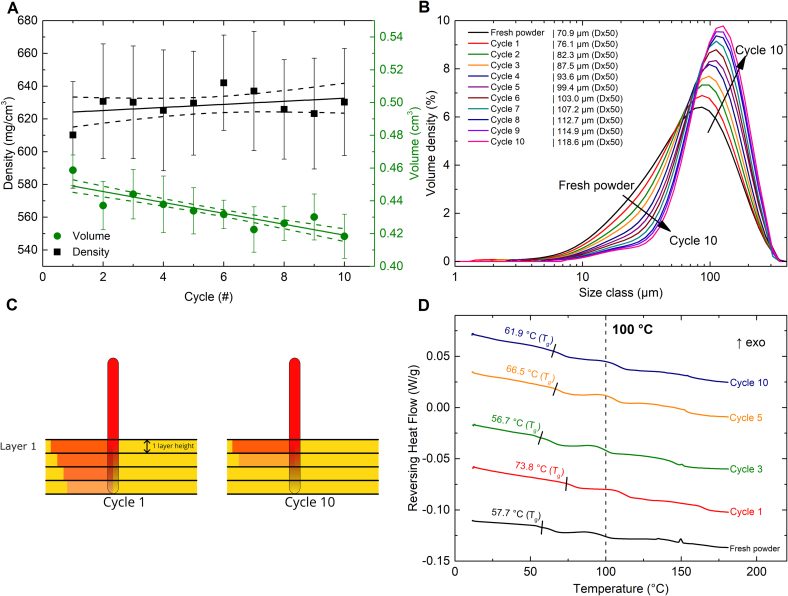
**A)** Tablet volume and density plotted over the ageing cycles. The line indicates the best linear fit, while the dashed line indicates the 95 % confidence interval. The individual values shown are the mean ± s (n = 8). **B)** The particle size distribution of the cycled PVPVA formulation. **C)** Differences in the degree of sintering between small (cycle 1) and large (cycle 10) particles with identical laser penetration depth. **D)** Thermographs obtained via mDSC of the PVPVA formulation over the ageing cycles, measured in the presence of moisture. The dashed line indicates the bed temperature, while the short black lines mark the onset of the T_g_.

The average tablet weight and tensile strength gradually decreased over the print cycles (Fig. 2A, B (green)). The reduction in tablet weight is primarily due to a significant decrease in tablet volume over the cycles (*p* < 0.0001), while the tablet density shows no significant change (*p* = 0.28). The *p*-values were determined using simple linear regression (Fig. 4A). As the diameter of the tablets remained constant over the cycles (10.25 ± 0.06 mm in cycle 1 and 10.18 ± 0.12 mm in cycle 10), the height must be the determining factor. Indeed, the tablet height in cycle 1 was 5.56 ± 0.12 mm and the average tablet height in cycle 10 was 5.14 ± 0.09 mm, while the designed tablet height was only 4 mm. The discrepancy between the designed and observed tablet height can be explained by the heat penetration from the illuminated top layer into the underlying powder bed. As a consequence, several underlying powder layers are fused to the tablet. The decrease in tablet height over the cycles indicates that the laser energy fuses less powder to the base of the tablet over the cycles. This can be explained by the observed increase in powder particle size (Fig. 4B). According to Herring's scaling law (Eq. S2), larger particles have lower effective surface energy than smaller ones (Herring, 1950; Kang, 2005; Song et al., 1984).

Powders with smaller particles and therefore higher effective surface energy, like fresh or slightly aged powder, fuse more effectively under constant energy input, especially in the first layers where the laser fuses material at the tablet's base (Fig. 4C). As the laser penetrates deeper, its power diminishes, limiting fusion to smaller particles. Consequently, fresh powder with smaller particles enables greater growth at the tablet's base compared to recycled powder with larger particles, resulting in thinner and lighter tablets as particle size increases.

Based on the general decrease in the degree of sintering, a reduction in tablet tensile strength is also expected, which matches the experimental observation (Fig. 2B, Green). The particle size increase also led to improved powder flow as seen in **Fig. S4B**.

The increase in particle size is most likely a direct result of the residual powder slowly sintering together under the printing conditions. Under dry conditions, the bed temperature does not exceed the polymer's glass transition temperature (T_g_ = 104 °C). The PVPA formulation was not completely dry, however, and contained between 1.5 and 2.1 % moisture over the cycles. The presence of the water, a strong plasticizer, reduced the T_g_ below the bed temperature, as shown in Fig. 4D. Therefore, part of the material can soften and begin to sinter, i.e., fuse or cake together at the process temperatures. This sintering or caking process is slower than the sintering upon laser irradiation as the temperature remains close to the T_g_. During printing, the residual powder on the build plate slowly sintered together, as can be seen in **Fig. S7.** The material was manually broken up and sieved over a 315 μm to prepare it for the next print cycle.

The increase in particle size for the PVPVA formulation and the accompanied gradual decrease in tablet weight and tensile strength are undesirable ageing phenomena. The print conditions used in the current study were optimized for printing with fresh material. The cycling experiment shows that these printing conditions lead to poor recyclability of the material. It is advisable to print at lower temperatures to minimize or prevent the fusion of residual powder, thereby ensuring more consistent tablet weights.

#### 3.1.3 Methacrylic acid-ethyl acrylate (MAEA) copolymer

Ten print cycles of the MAEA-based formulations were successfully printed. After an initial decrease in tablet weight from 169.8 ± 4.3 mg in cycle 1 to 156.04 ± 4.4 mg in cycle 2, the tablet weight remained constant (Fig. 2A, black).

**Fig. 5:**
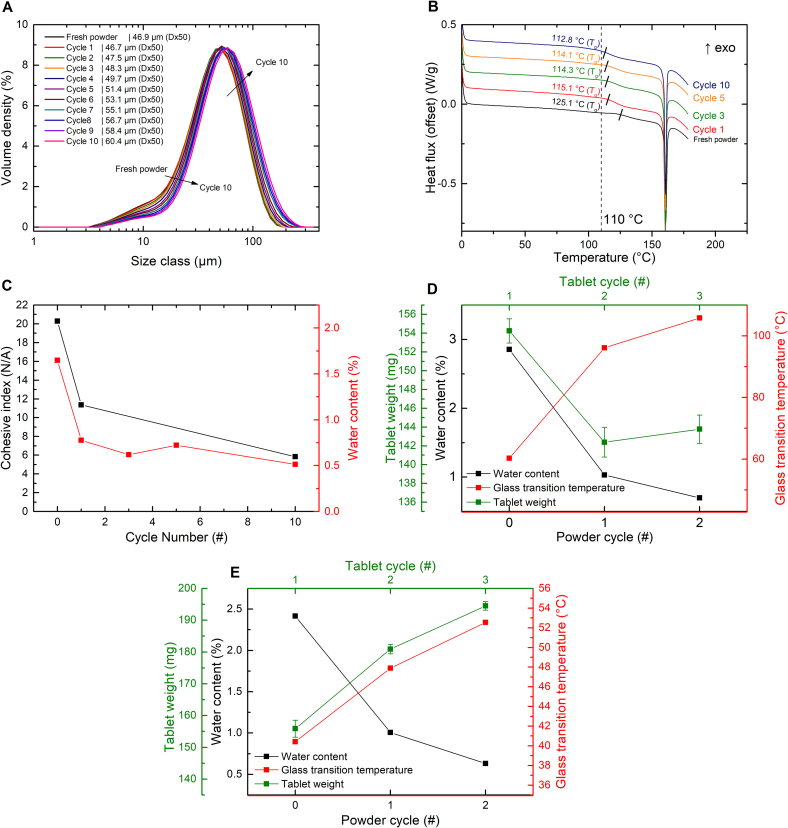
**A)** The particle size distribution of the cycled MAEA formulation. **B)** DSC thermographs of the MAEA formulation over the ageing cycles measured in the absence of moisture. **C)** Cohesiveness index at 2 rpm (data extracted from **Fig. S4C**) and water content (data extracted from **Fig. S5C**) over the ageing cycles for the MAEA formulation. The full datasets are provided in the corresponding Supplementary Information figures, while only the relevant portions are displayed here for clarity. **D, E)** Tablet weight, moisture content (measured on TGA Q50), and onset temperature of the MAEA (**D**) and PVA (**E**) formulation without indomethacin over three ageing cycles. Note that the water content and T_g_ follow the bottom x-axis while the tablet weight follows the top x-axis. This is done to clearly show the relation between the powder properties and the tablets that result from these properties. The glass transitions were measured with modulated DSC in sealed pans to retain moisture during the measurement.

The mean and standard deviation of all tablet weights from MAEA cycles 2–10 were only 154.5 ± 3.3 % (Fig. 2A, black). A small sign of powder ageing is that the standard deviation of the tablet weight is gradually increasing each cycle from 2.5 % at cycle 2 to 3.9 % at cycle 10. This can be caused by a slight increase in particle size (Fig. 5A) and minor deviations in the polymer structure as indicated by the slight changes in T_g_ (Fig. 5B).

The initial decrease in tablet weight (Fig. 2A, black) is attributed to a decrease in tablet density (Fig. 2D, black). This change is accompanied by a reduction in both moisture content and cohesive index (Fig. 5C), similar to what was observed for the PVA formulation (Fig. 3A).

To better understand the role of moisture on tablet weight, the cycling experiment was repeated for three cycles with both the PVA and MAEA formulations, this time excluding indomethacin. Results shown in Fig. 5D and Fig. 5E confirm that the initial change in tablet weight from cycle 1 to cycle 2 is reproducible for both formulations and consistent with earlier findings (see Fig. 2, black and orange). The initial change in tablet weight is accompanied by a drop in moisture content for both formulations. Importantly, the data shows an inverse correlation between moisture content and T_g_. This is expected, as water is a strong plasticizer, which is a well-established phenomenon for the polymers used in this study (Li et al., 2021; Palzer, 2005; Patel et al., 2023; Patel and Serajuddin, 2022). The Gordon-Taylor equation (Eq. S3) also describes this relationship (Gordon and Taylor, 1952; Sablani et al., 2007).

When the T_g_ of a polymer is reduced and gets closer or even below the bed temperature (T), powder cohesion tends to rise (Fitzpatrick et al., 2007; Foster et al., 2006; Palzer, 2005). Therefore, a decrease in moisture content resulting in a higher T_g_, leads to lower powder cohesion. This aligns with the observed decreases in cohesive index for both formulations (Fig. 3A and Fig. 5C).

For the MAEA formulation, these changes in cohesive index did not visibly affect the powder layer quality. MAEA's cohesive index remained below 25 throughout, a value generally considered suitable for good layer spreading (Baesso et al., 2021; Understanding and Improving Powders Spreadability For a Recoater Process in Additive Manufacturing, 2020). The reason that the tablet weight decreased after the first cycle can be explained using the Williams-Landel-Ferry equation (Eq. S4), which shows that a lower T_g_ (due to higher moisture content) results in lower melt viscosity at a given processing temperature (Williams et al., 1955). Lower melt viscosities promote better coalescence and densification. As a result, for MAEA, tablet weight has a negative correlation with T_g_ (Fig. 5D). An increased T_g_ increases the melt viscosity and therefore reduces the tablet weight.

For PVA, the reduction in moisture content after the first print cycle led to a visible improvement of the poor powder spreadability (Fig. 3B**)**, resulting in denser powder layers and therefore heavier tablets after the first cycle. It is hypothesized that the improvement in powder flow had a stronger influence on tablet weight than the increase in T_g_ (Fig. 5E) and the corresponding increase in melt viscosity. The effect of moisture content on powder cohesion and melt viscosity could be further explored using melt rheology and additional powder flowability measurements at elevated temperatures.

The initial change in tablet weight for both the PVA and MAEA formulation is undesirable but can be avoided by preheating the material to establish a steady printing state. However, static preheating does not suffice, as the material forms a cohesive cake that is difficult to de-aggregate. To prevent this, the material should be continuously agitated during preheating, ensuring it remains free-flowing and ready for printing. Monitoring a formulation's moisture content is critical in assuring consistent tablet quality.

The MAEA performed consistently under the tested cycling conditions after the initial change in powder and tablet properties, the material remained similar. The recycling experiment was stopped after 10 cycles, but there was no indication that the printing performance of the materials had lowered. The overall tensile strength, however, is low with approximately 0.3 MPa. To increase the mechanical strength of the tablet, a higher laser energy density could potentially be applied at higher printing temperatures. But these changes in print parameters could affect the recyclability of the material.

#### 3.1.4 Powder refreshment

Powder refreshment is commonly performed to retain material volume, recycle material, and remedy ageing effects. After ten print cycles or after print failure, the formulations were refreshed with 50 wt% fresh powder to assess if the aged material could be remedied and if the weight of the obtained dosage forms returned to its initial values.

**Fig. 6:**
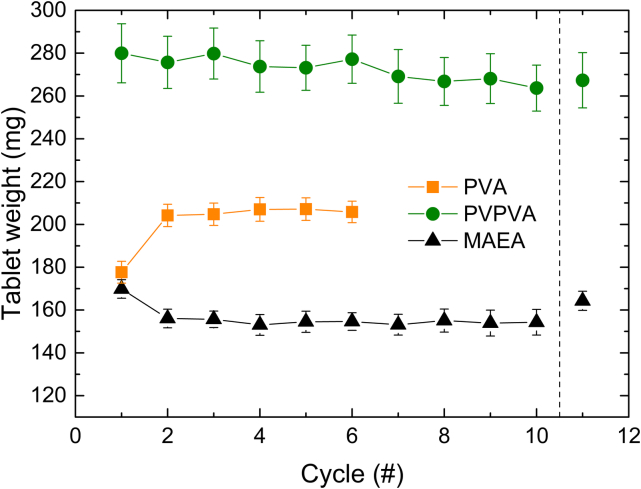
The average tablet weight for the PVA, PVPVA, and MAEA over the printing cycles, including the 11th cycle with 50 % refreshed material printed with the SnowWhite^2^ printer.

After the addition of 50 % fresh powder, the PVA formulation remained unprintable (Fig. 6, orange). It was not investigated if starting earlier with refreshing the PVA formulation would have led to increased cyclability. The PVPVA formulation showed a small but non-significant increase in tablet weight (Fig. 6, green). If the material is refreshed after every cycle, potentially, a consistent printing quality could be achieved. The MAEA formulation showed a significant increase in weight (Fig. 6, black). The increase in tablet weight for the MAEA is undesirable as it moves away from the steady printing state of the aged material. Still, a constant refreshing of pre-heated (dried) MAEA may also lead to constant tablet quality attributes.

Refreshing the formulation is not necessary to improve print quality for the PVA and MAEA under the print conditions used in SnowWhite^2^. Yet, it remains necessary to blend in fresh powder to compensate for the powder that is printed into tablets to keep a sufficient powder volume for the next production batch. For these materials, formulation refreshment must be done with preconditioned powder to ensure a free-flowing powder with a consistent moisture content.

### 3.2 Printer hardware considerations

The printers are challenging to compare directly as the printing parameters are difficult to translate from one printer to the other, given their different heating and laser systems. For comparison purposes, the second print cycle was chosen as most formulations reached a steady printing state only after the first cycle. The variation around the average tablet weights for the second cycle prints of all three formulations on the SnowWhite^2^ are lower compared to the second cycle prints from the Kit, as shown in Table 4**.**

**Table 4:** Standard deviations around the average tablet weight for the different formulations printed in the second cycle on the SnowWhite^2^ and Kit (n = 32, * n = 18).

	SnowWhite^2^	Kit
PVA	2.6 %	3.7 %
PVPVA	4.4 %^⁎^	5.1 %
MAEA	2.8 %	7.5 %

The Kit printer, however, was able to print the PVPVA formulation with the 32 tablets, whereas the SnowWhite^2^ printer was only able to do a narrower 18-tablet pattern. The halogen lamp heating system in the SnowWhite^2,^ in combination with poor insulation around the build platform, is hypothesized to result in a temperature gradient on the surface of the build platform with a hot spot in the center and a colder region on the edges of the build platform. The temperature gradient leads to difficulties with printing parameter optimization as the outside tablets experience lower process temperatures than the inside tablets. The Kit printer has resistive heating at the back of the heated bed in addition to the halogen lamps heating from the top. The addition of resistive heating at the back of the heating bed can potentially lead to a more evenly heated bed. Making it possible to print closer to the edge of the build platform, which allows the 32-tablet pattern to be printable. The additional heating, however, also exposes the residual powder to elevated temperatures for prolonged periods, potentially leading to an increase in powder ageing. This increased energy input by prolonged heat exposure may be the cause of the increased data variations. An alternative to resistive heating would be improved heat insulation around the sides of the powder bed.

Another major difference between the printers is the laser wavelength, where the SnowWhite^2^ uses a CO_2_ laser with a wavelength of 10.64 μm while the Kit uses a blue diode laser with a wavelength of 445 nm. Using a CO_2_ laser has the advantage that no colorants are needed because organic molecules absorb the infrared laser light well. This leads to simplified formulations, exposing patients to fewer potentially harmful excipients. Still, this is a double-edged sword as it also means that energy absorption is difficult to tune, as almost all excipients and APIs will absorb at this wavelength. A blue diode laser in combination with a colorant in the formulation allows for fine control over the laser absorption through a change of colorant concentration in the formulation.

## 4 Conclusion

In this work, we investigated the ageing behavior of three pharmaceutical polymers during recycling in LPBF 3D printing. The PVA formulation can be successfully recycled for five cycles, while the MAEA formulation is found to be the most robust material, as it can be successfully recycled nine times without loss in performance. Recycling of the PVPVA formulation under the current SnowWhite2 print conditions resulted in significant performance loss and is therefore not recommended without further process optimization. Still, the results obtained with the SnowWhite^2^ printer show a strongly improved reproducibility compared to the results obtained with the Kit printer. Samples prepared on the Kit printer displayed large and random variations within print cycles as well as over the different print cycles, preventing proper interpretation of the data. The underlying reason for the variation could not be identified and is most likely process-related.

We found that powder ageing heavily depends on the print conditions under which the materials are printed. The impact of different print conditions on recyclability has not been evaluated in this work, but should be investigated in future experiments in detail, which could lead to additional insights.

The ageing phenomena observed in the studied pharmaceutical formulations are related to changes in particle size distribution, moisture content, glass transition temperature, and powder cohesion. The underlying ageing mechanisms are therefore fundamentally different than those for PA12. Unlike PA12, the ageing behavior of the studied pharmaceutical polymers does not always require full powder refreshing to maintain performance, provided sufficient powder volume is maintained. These findings challenge assumptions based on engineering polymers like PA12 and highlight the need for material-specific powder management strategies in pharmaceutical LPBF.

Powder ageing is a material and process-dependent phenomenon that significantly influences print performance. Considering powder ageing during formulation development is critical for optimal print quality, establishing sustainable process conditions, and advancing the pharmaceutical application of laser powder bed fusion.

## CRediT authorship contribution statement

**Wessel Kooijman:** Writing – review & editing, Writing – original draft, Methodology, Investigation, Formal analysis, Conceptualization. **Valerie R. Levine:** Writing – review & editing, Methodology, Investigation, Conceptualization. **Robbert J. Kok:** Writing – review & editing, Supervision. **Jonas Lindh:** Writing – review & editing, Supervision. **Julian Quodbach:** Writing – review & editing, Supervision, Project administration, Conceptualization.

## Declaration of competing interest

The authors have no conflict of interest to declare.

## Data Availability

Data will be made available on request.

## Appendix A Supplementary data

Supplementary material Image 1
