# Supplementary material for: The effects of recycling pharmaceutical formulations in laser powder bed fusion 3D printing - the influence of physical phenomena on printing performance
Source: Int J Pharm X. 2025 Aug 30;10:100383. doi: 10.1016/j.ijpx.2025.100383 (PMC12446654; doi:10.1016/j.ijpx.2025.100383)
Supplement: Supplementary file 1 — Supplementary material [file mmc1.docx]

**Supporting information**

The effects of recycling pharmaceutical formulations in Laser powder bed fusion 3D printing

The influence of physical phenomena on printing performance

Wessel Kooijman ^a^, Valerie R. Levine ^b^, Robbert J. Kok ^a^, Jonas Lindh ^b^, Julian Quodbach ^a,*^

^a^ Division of Pharmaceutics, Utrecht Institute for Pharmaceutical Sciences (UIPS), Utrecht University, Universiteitsweg 99 3584 CG, Utrecht, the Netherlands.

^b^ Division of Nanotechnology and Functional Materials, Department of Material Science and Engineering, Uppsala University, Uppsala SE-751 03, Box 35, Sweden

^*^ corresponding author.

E-mail address: j.h.j.quodbach@uu.nl

**Supporting information.**

**Section 1** provides additional characterization data of the studied pharmaceutical formulations that were not included in the main manuscript. The experiments are presented in the order mentioned in the text. Experimental methods are described in the main manuscript. Additionally, photographic images of visual observations are included at the end of this section.

**Section 2** contains a detailed analysis of the results obtained with the Sintratec Kit printer, following a similar approach to that used for the SnowWhite2 printer. As noted in the main text, samples printed using the Sintratec Kit exhibited large and inconsistent variations both within and between print cycles, hindering meaningful interpretation of the data.

**Section 3** presents additional characterization of the materials obtained from the printing cycles performed with the Kit printer.

**Section 4** lists the three equations referenced in the main text.

1. Analytical data SnowWhite^2^

**X-ray powder diffraction** (XRPD)

The X-ray powder diffractograms of samples from all formulations printed using the SnowWhite^2^ are shown below.










**Fig. S1** X-ray powder diffractograms of the PVA (A), PVPVA (B), and MAEA (C) formulations taken over the ageing cycles performed in the SnowWhite^2^ printer. These diffractograms were measured using Co Kα radiation.

Fourier transformed infra-red spectroscopy (FTIR)

The FTIR spectra of samples from all formulations printed using the SnowWhite^2^ are shown below. The wavelength of the CO_2_ laser utilized in the SnowWhite^2^ has been indicated with a black dashed line.

**
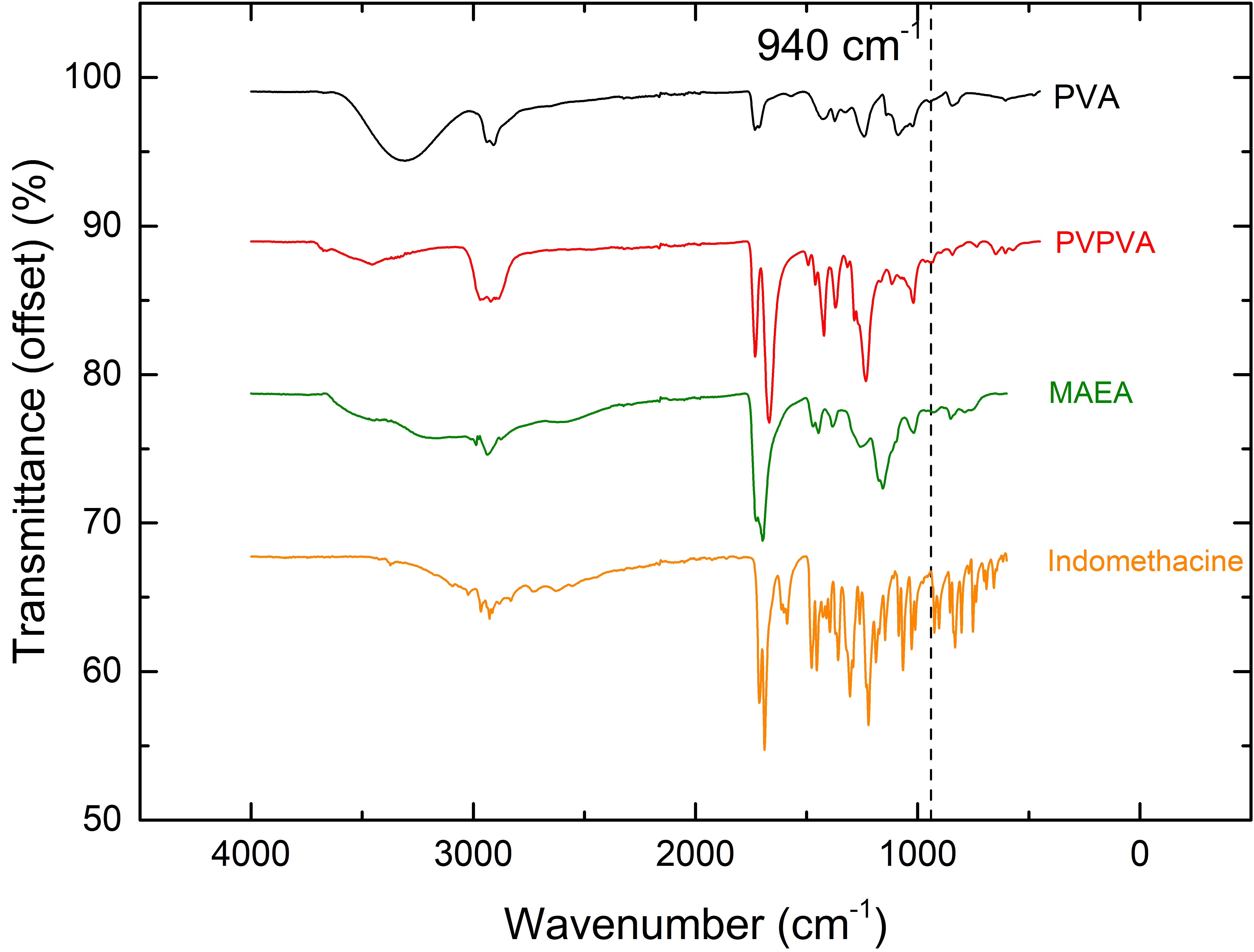
**

**Fig. S2** FTIR Spectra of the thermoplastic polymer and indomethacin used in the studied formulation. The dashed line indicates the wavenumber at which the CO_2_ laser used in the SnowWhite^2^ printer operates.

**
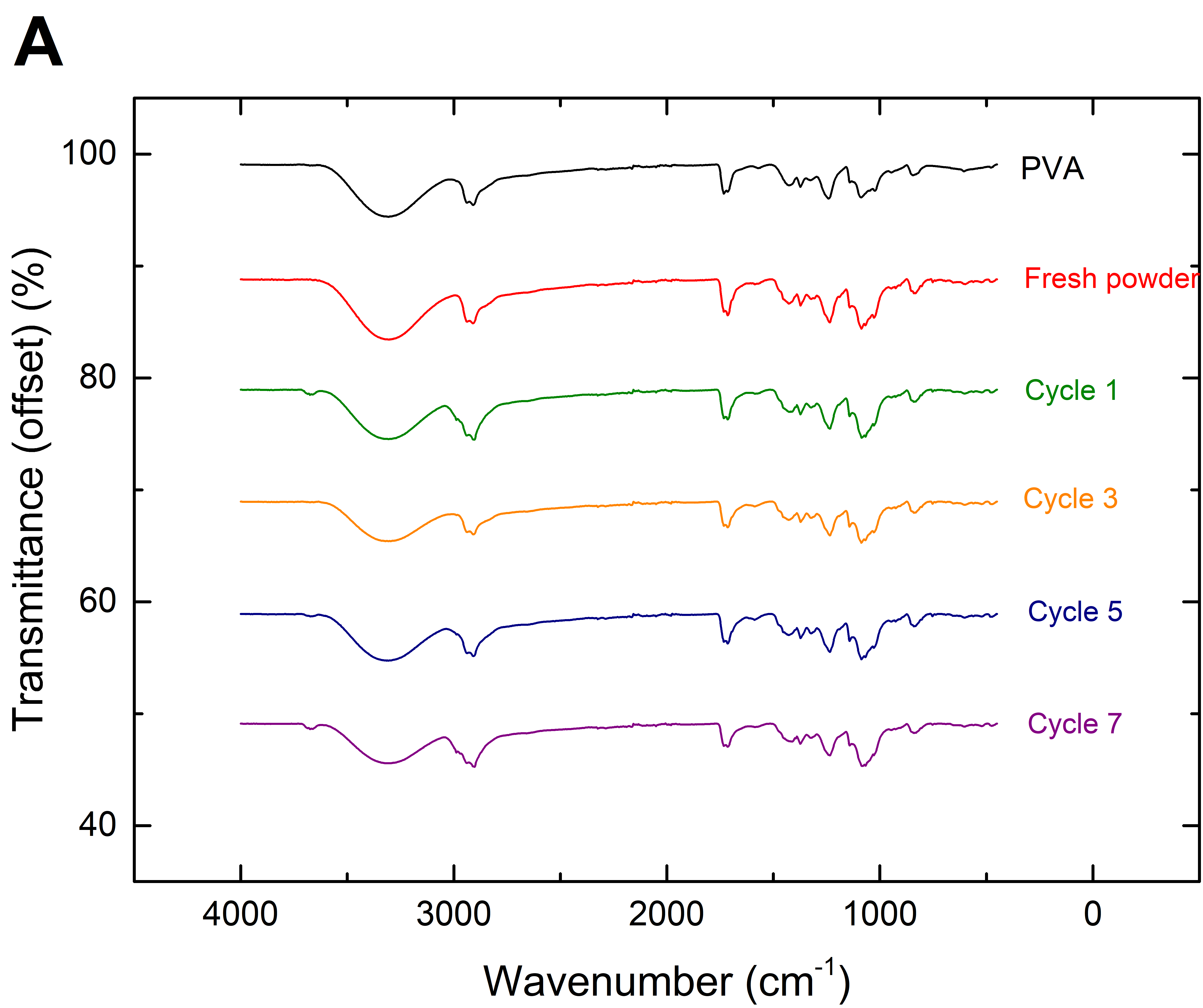

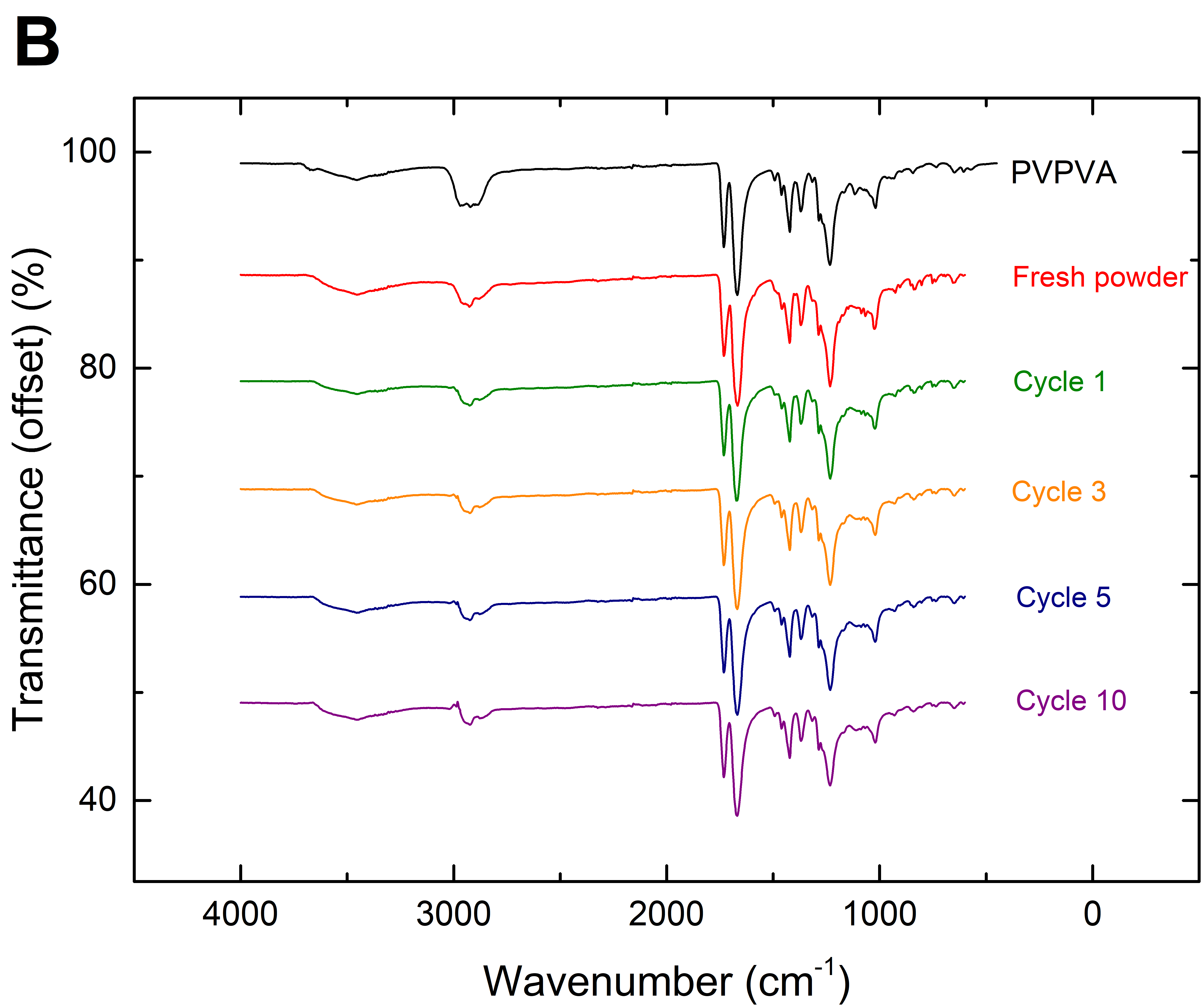

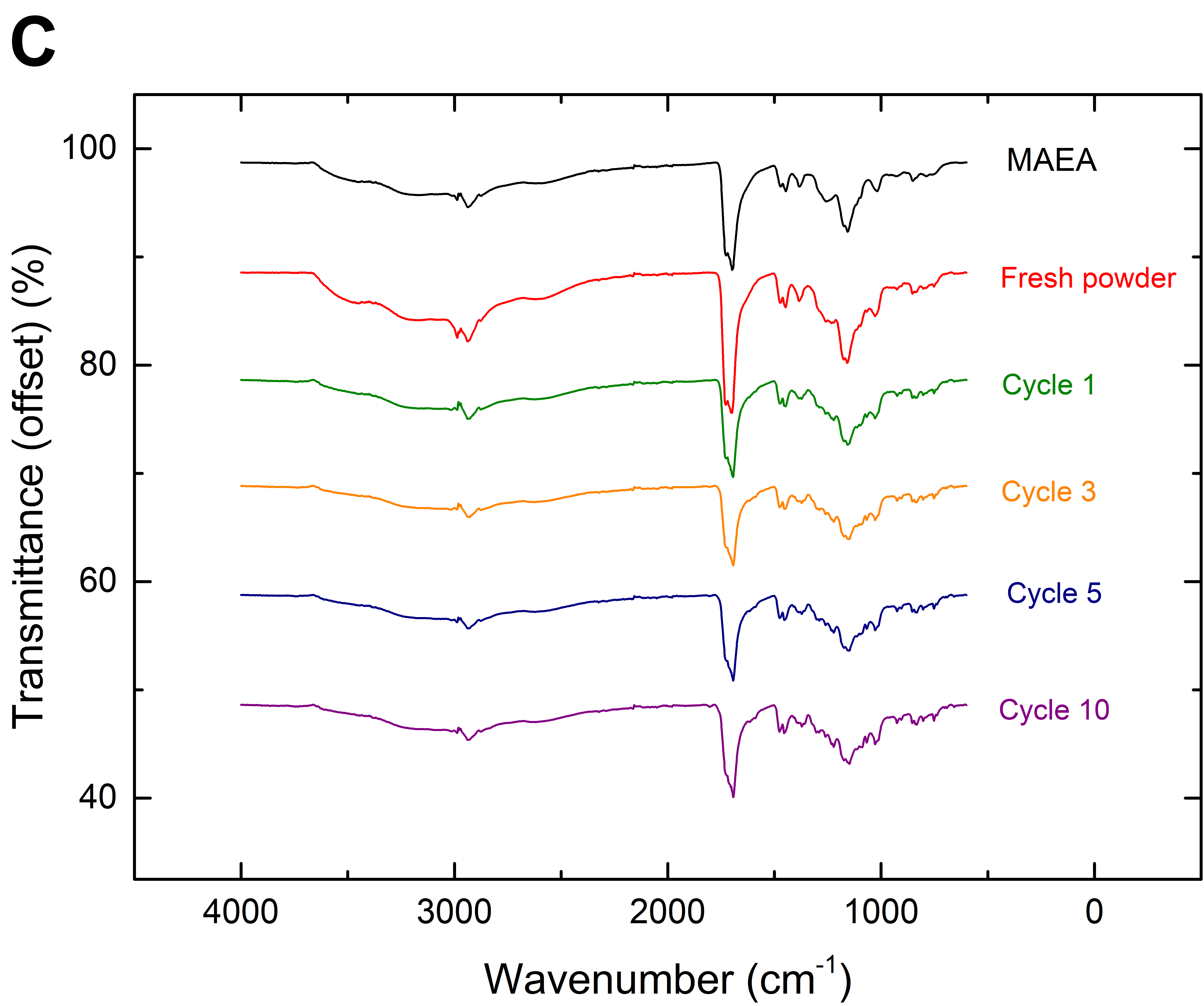
**

**Fig. S3** FTIR spectra of the PVA (A), PVPVA (B), and MAEA (C) formulations taken over the ageing cycles performed in the SnowWhite^2^ printer.

Powder rheology

The granudrum data of samples from all materials printed using the SnowWhite^2^ are shown below.


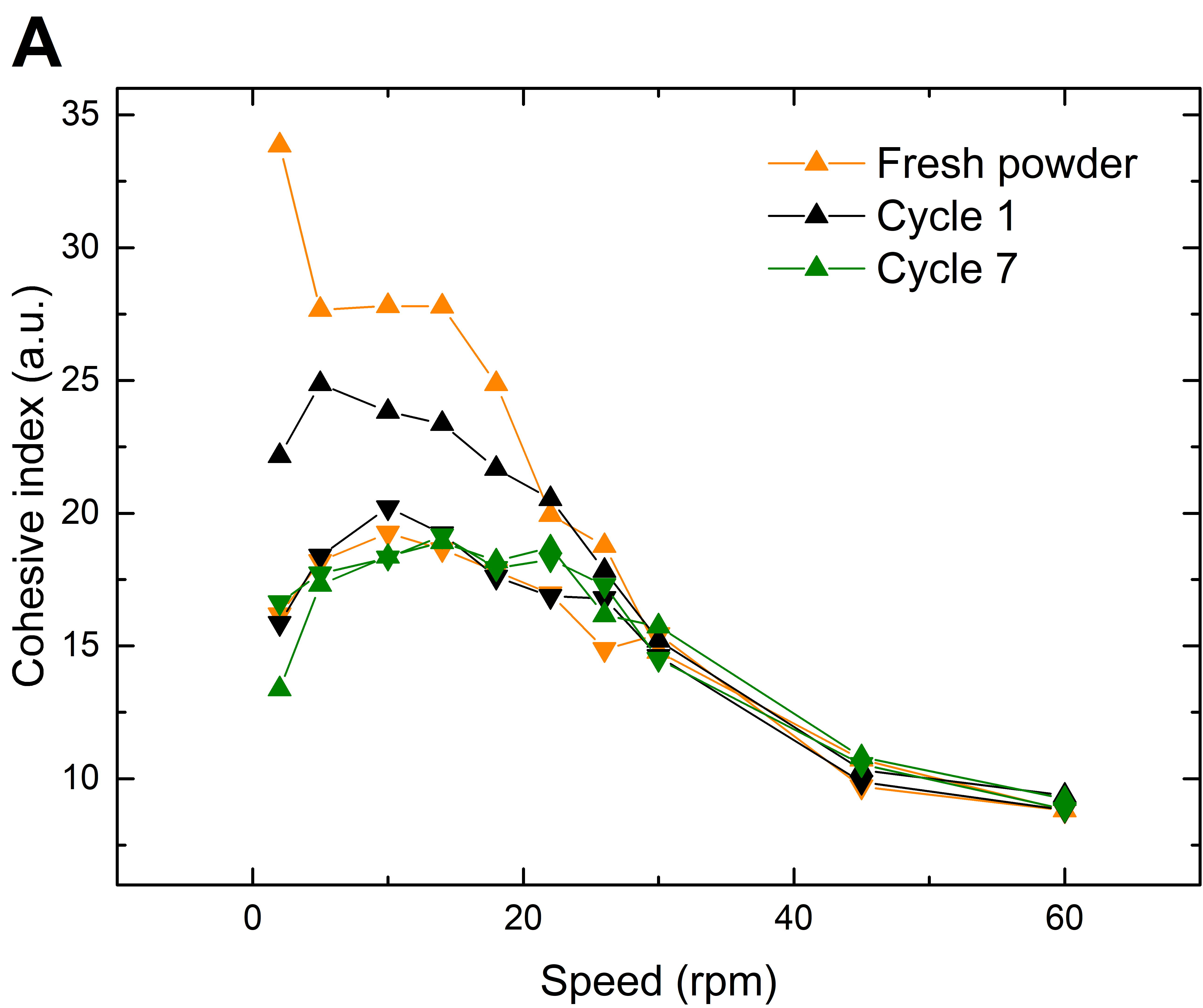

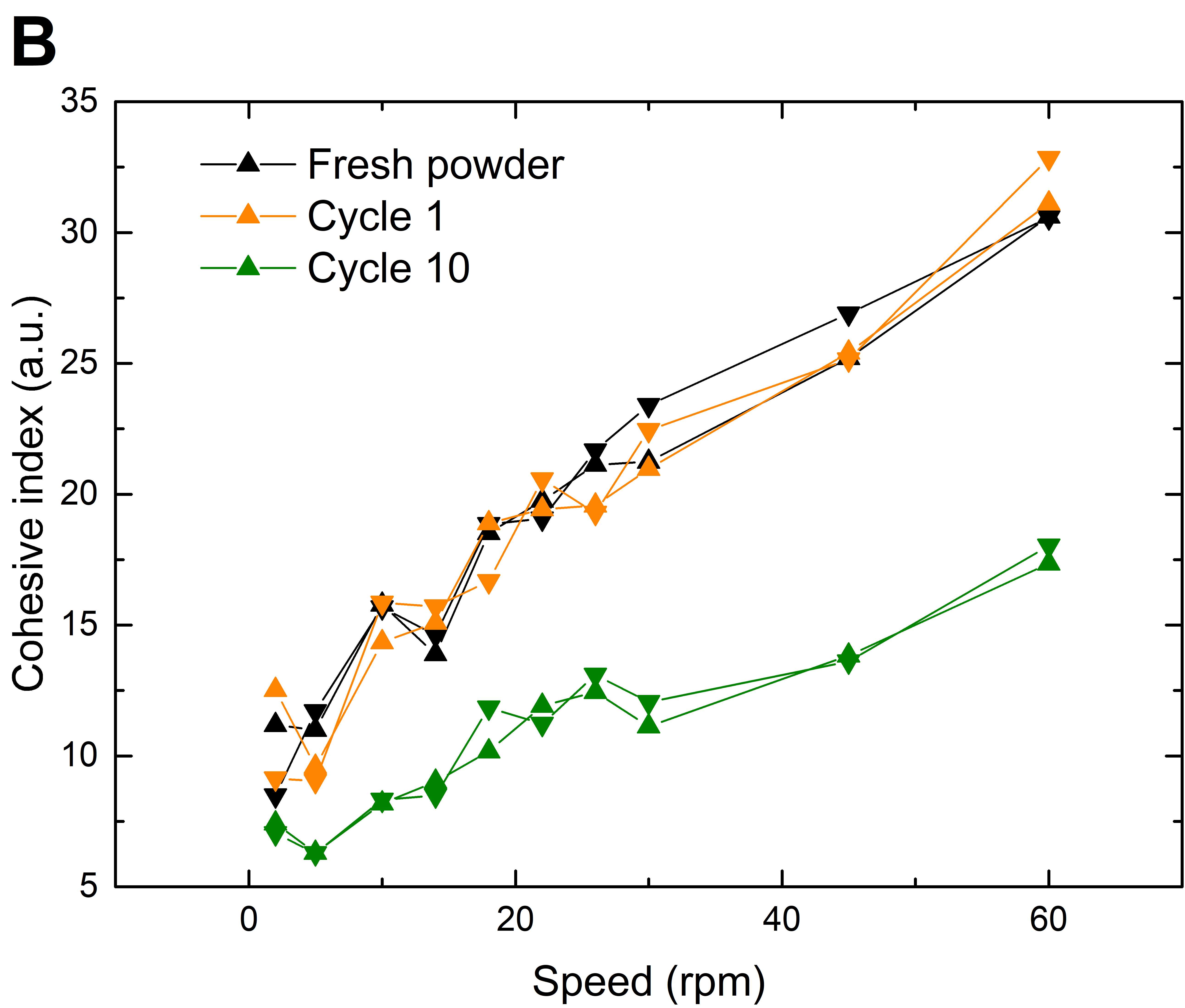


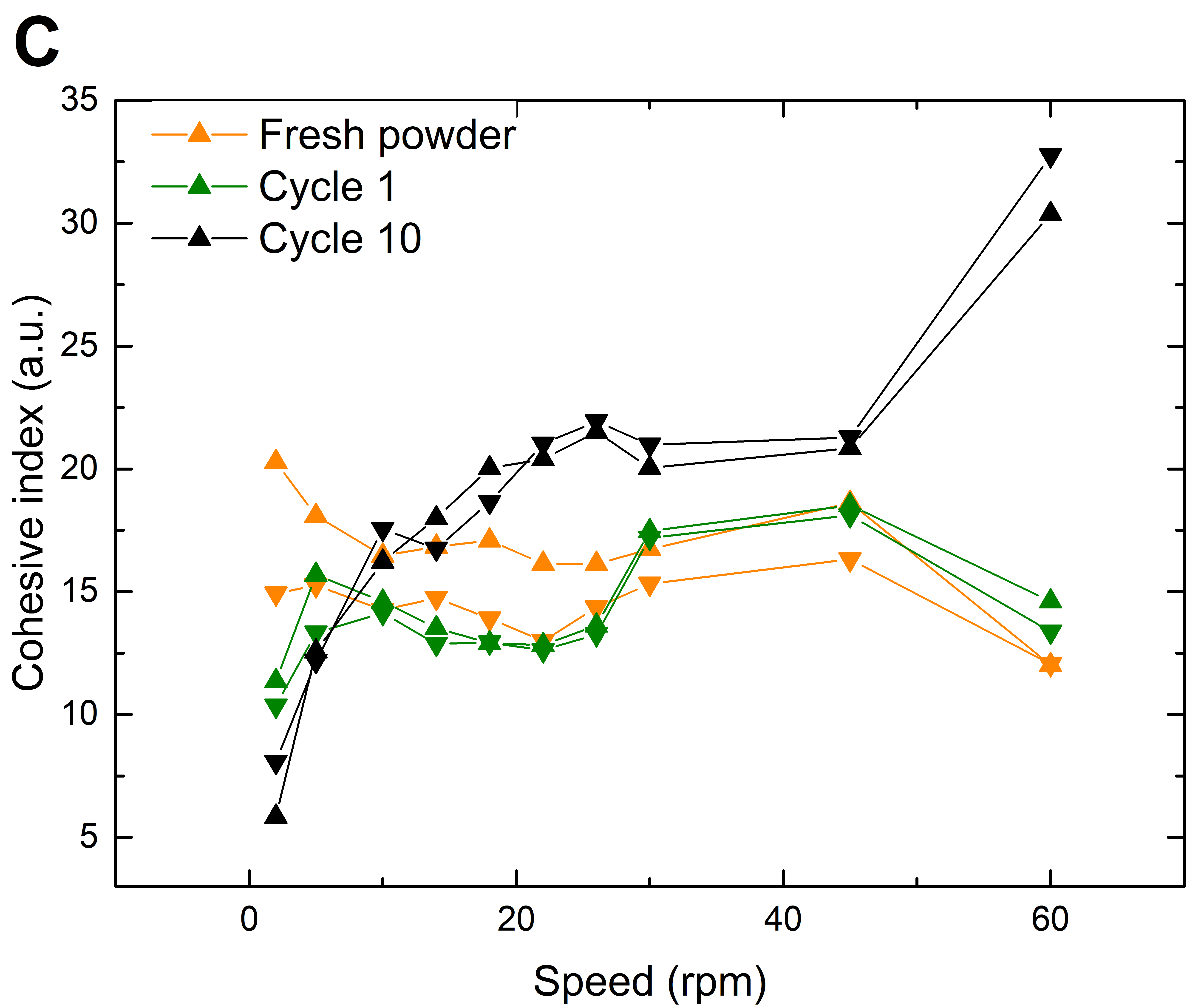


**Fig. S4** Cohesive index at different rotational speeds for the PVA (A), PVPVA (B), and MAEA (C) based formulations taken over the ageing cycles performed in the SnowWhite^2^ printer. The triangles pointing up indicate the acceleration sequence, while the triangles pointing down indicate the deceleration sequence of the same experiment.

Thermogravimetric analysis (TGA)

The TGA graphs of samples from all formulations printed using the SnowWhite^2^ are shown below.


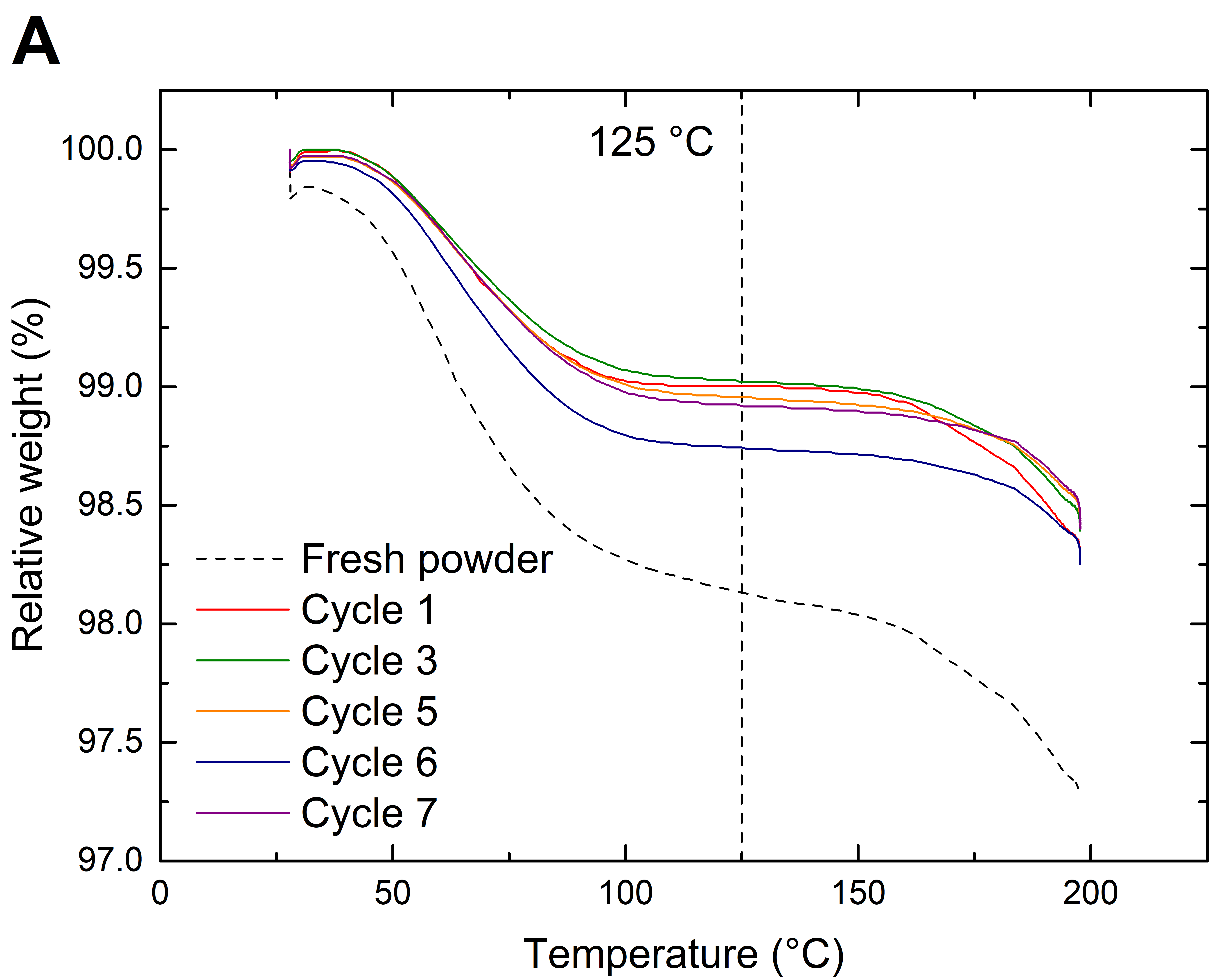


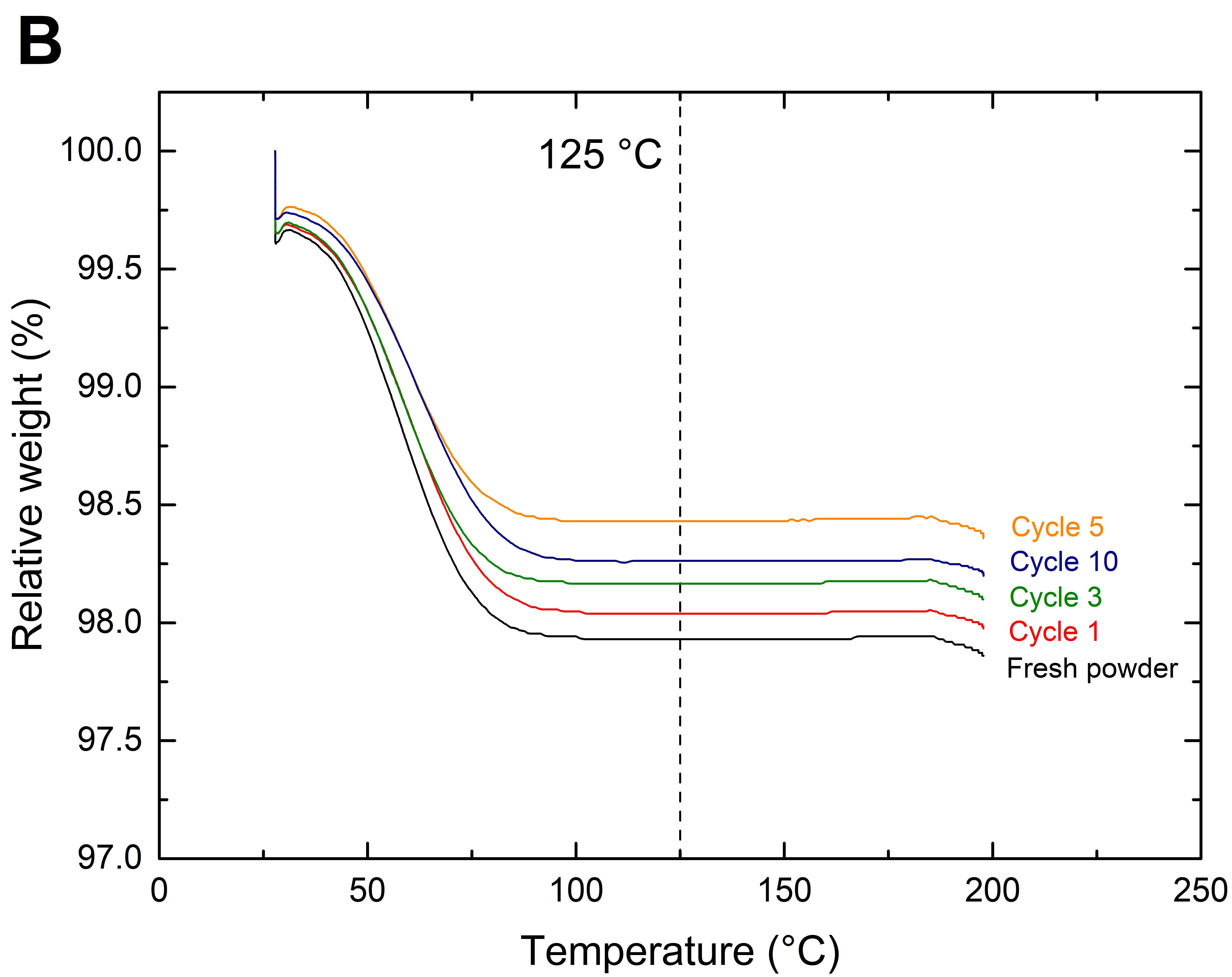


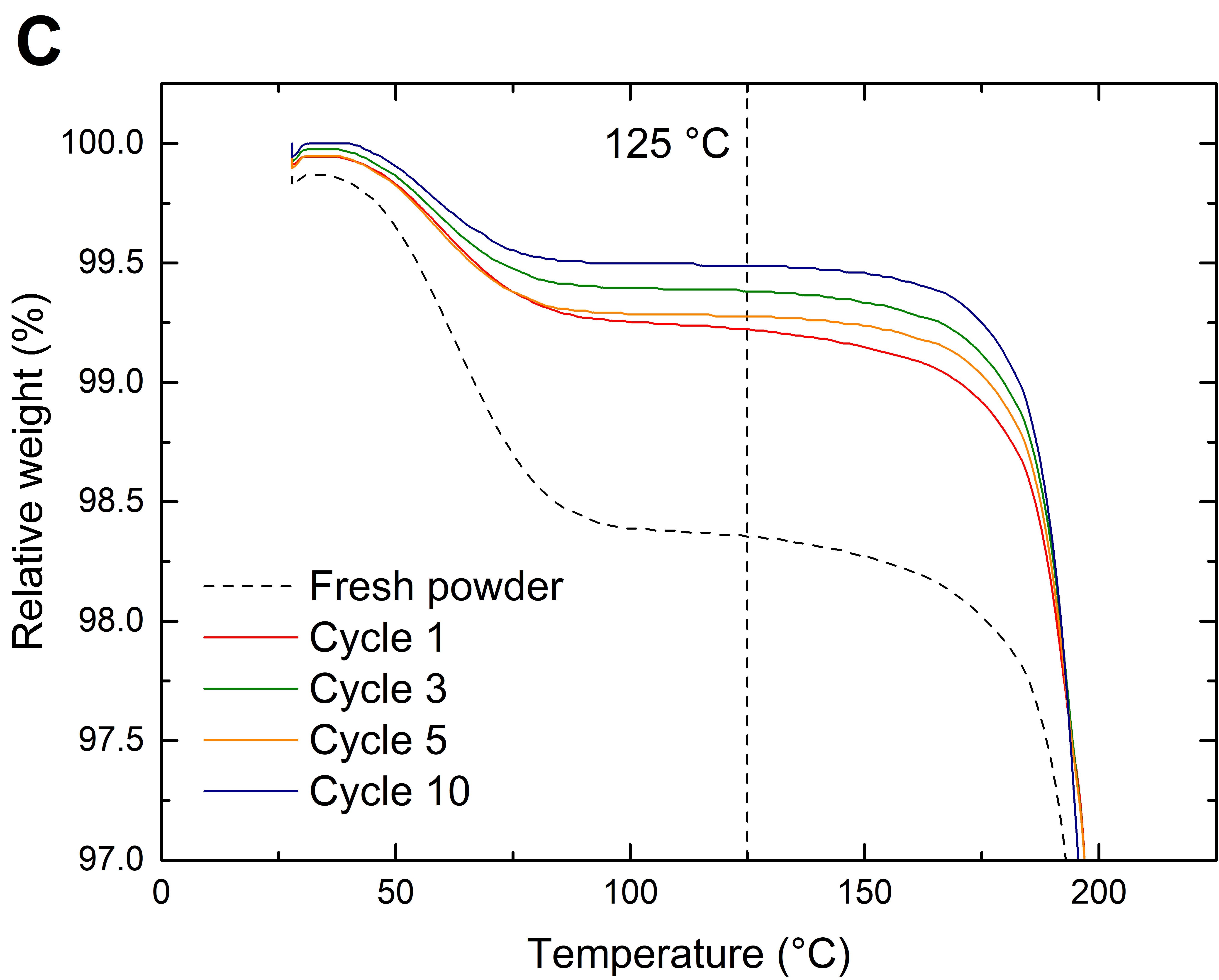


**Fig. S5** *TGA thermographs of the PVA (A), PVPVA (B), and MAEA (C) formulation taken over the ageing cycles performed in the SnowWhite^2^ printer. The dashed line indicates the temperature at which the relative weight loss was determined as loss on drying. Measurements were performed on the TGA/DSC 3+.*

Spatial tablet weight

For the PVPVA tablets, the tablet weight for each tablet position in the print bed was registered. This was possible because the residual powder surrounding the tablets formed a cake, which kept the tablets in place upon extraction of the tablets from the printer.


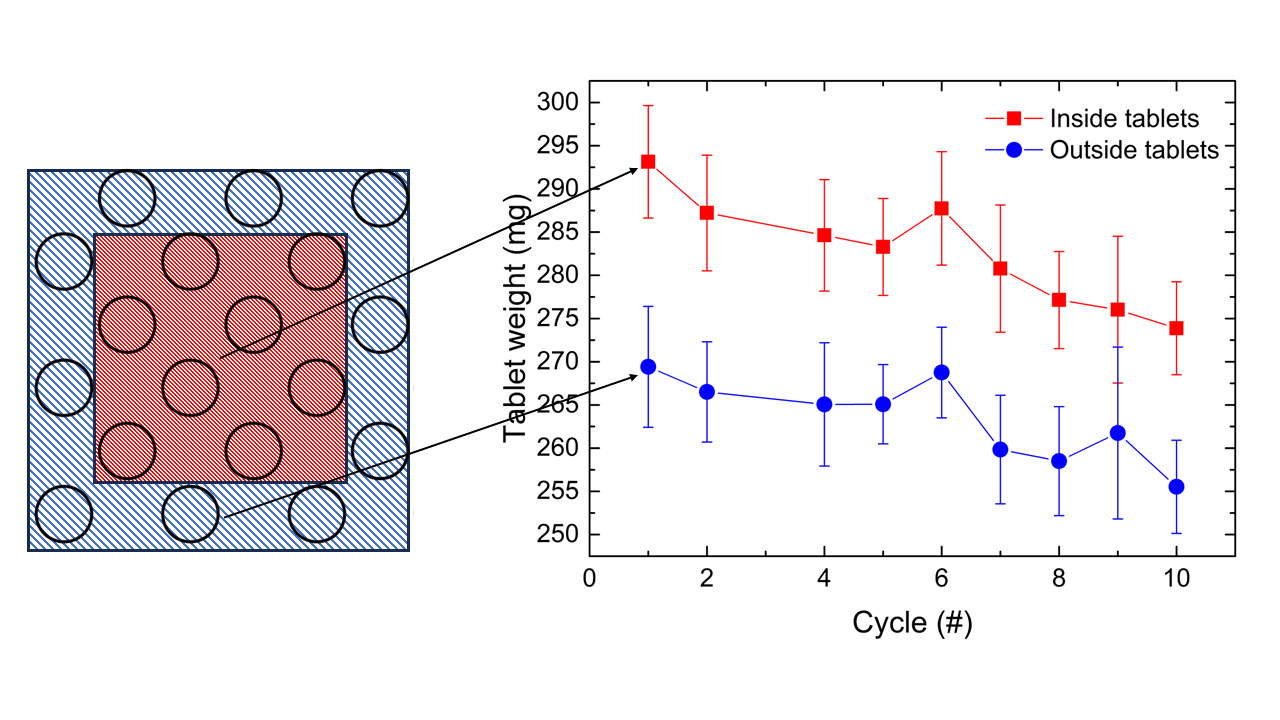


**Fig. S6** *Tablet weight over the PVPVA printing cycles, split into the weight of the outside tablets and inside tablets, as indicated by the diagram. The individual values shown are the mean ± s.*

Miscellaneous images

During the process, some specific observations were made, which were captured in the image shown below.


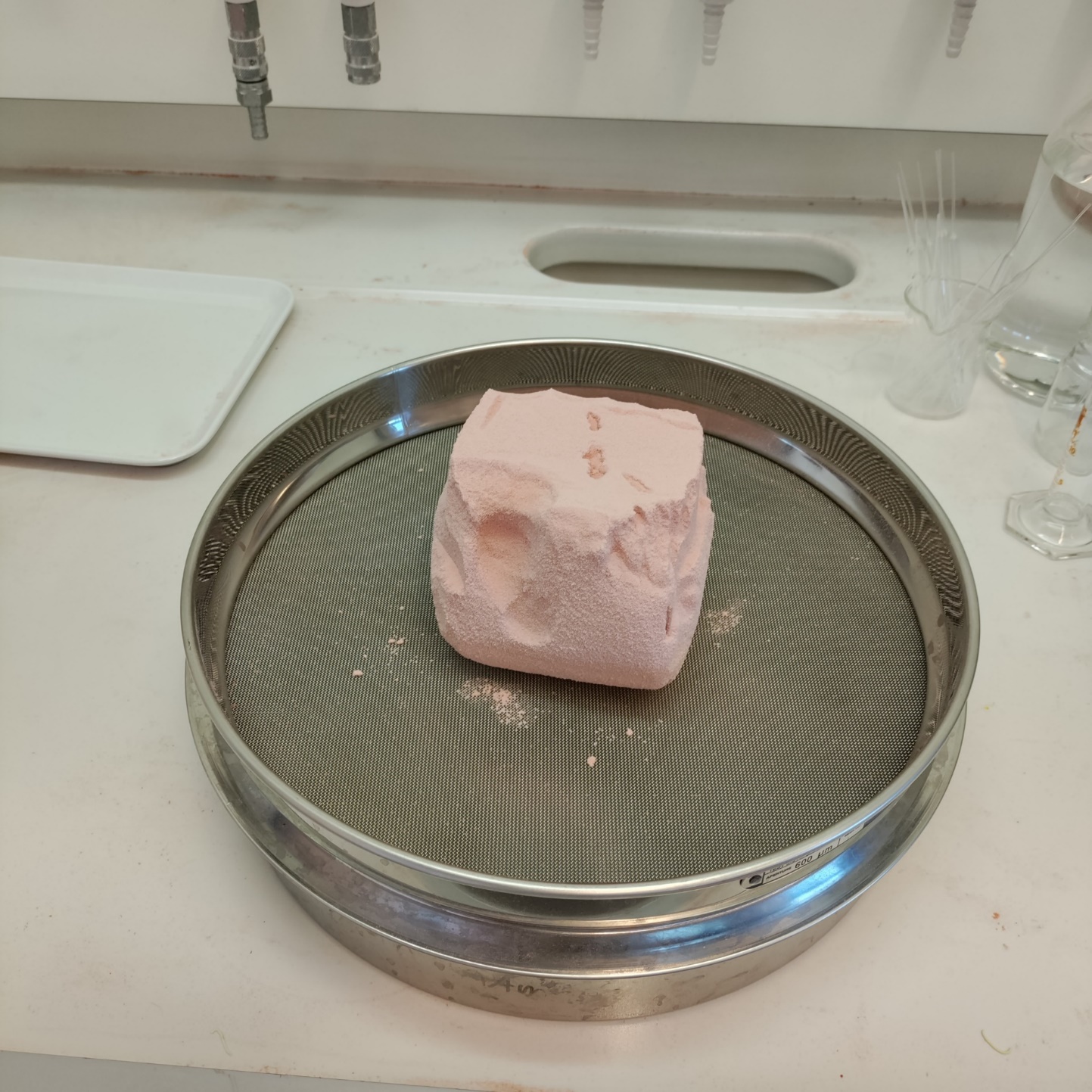


**10 cm**

**Fig. S7** Material obtained from the SnowWhite^2^ printer after every printing cycle with the PVPVA formulation.

Differential scanning calorimetry (DSC)

The DSC thermographs of the PVA and MAEA samples obtained from the SnowWhite^2^ can be found in the results and discussion section of the main text. The DSC thermograms from samples obtained with the PVPVA formulation printed on the SnowWhite^2^. The Glass transition temperatures for all 3 formulations printed on the SnowWhite^2^ in **Table S1**.

**Table S1**

The Glass transition temperatures for all 3 formulations printed on the SnowWhite^2^ and Sintratec kit printer.

|  | SnowWhite^2^ | | |
| --- | --- | --- | --- |
| Cycle | PVA | PVPVA | MAEA |
|  | T_g_ (°C) | T_g_ (°C) | T_g_ (°C) |
| 1 | 57.4 | 104.3 | 125.1 |
| 2 | 59.2 | 104.3 | 115.2 |
| 3 | 59.8 | 101.6 | 116.0 |
| 4 | 60.3 | 104.7 | 114.3 |
| 5 | 61.4 | 104.3 | 114.1 |
| 6 | 61.4 | 103.6 | 114.1 |
| 7 | 61.9 | 108.0 | 114.0 |
| 8 |  | 103.5 | 112.4 |
| 9 |  | 105.8 | 112.1 |
| 10 |  | 105.9 | 112.8 |


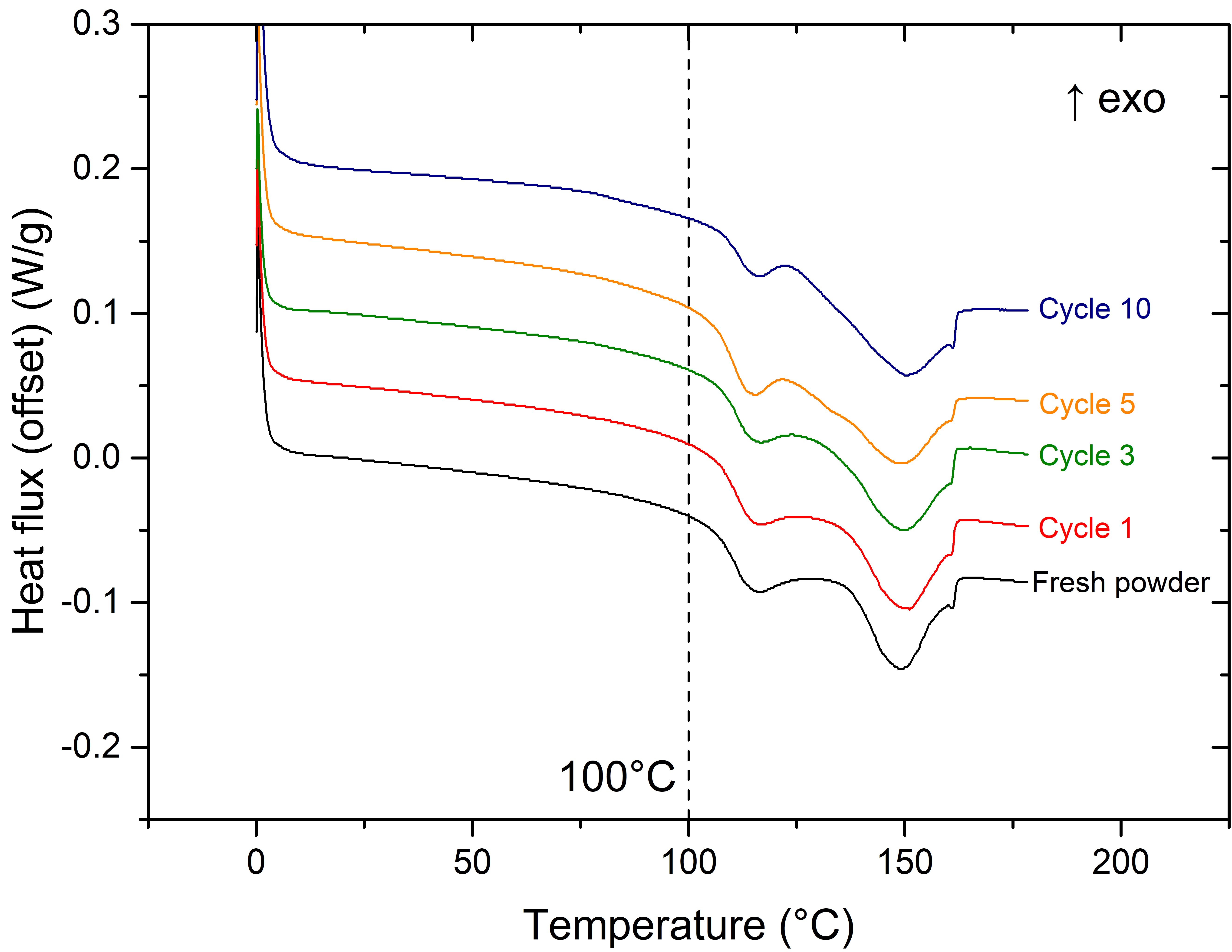


**Fig. S8** *DSC thermographs of the PVPVA formulation taken over the ageing cycles performed in the SnowWhite^2^ printer. Samples were measured in the absence of moisture.*

Particle size distribution

The particle size distributions obtained from the samples printed in the SnowWhite^2^ are shown in the main text. The Dx10, Dx50, Dx90, and the span of the particle distribution are shown in **Table S2** for the 3 different formulations printed with the SnowWhite^2^ printer. The span is calculated with the following equation:

$$\begin{aligned} Span= \frac{\left( Dx90-Dx10 \right)}{Dx50} \#\left( S SEQ EQUATION 1 \right) \end{aligned}$$

**Table S2**

The Dx10, Dx50, Dx90 and the span of the particle size distributions obtained from the cycled formulations in the SnowWhite^2^.

|  | **PVA** | | | | **PVPVA** | | | | **MAEA** | | | |
| --- | --- | --- | --- | --- | --- | --- | --- | --- | --- | --- | --- | --- |
| **Cycle (#)** | **Dx10**  **(µm)** | **Dx50**  **(µm)** | **Dx90**  **(µm)** | **Span** | **Dx10**  **(µm)** | **Dx50**  **(µm)** | **Dx90**  **(µm)** | **Span** | **Dx10**  **(µm)** | **Dx50**  **(µm)** | **Dx90**  **(µm)** | **Span** |
| **Fresh** | 14.3 | 46.5 | 112.9 | 2.12 | 19.9 | 70.9 | 172.9 | 2.16 | 17.2 | 46.9 | 91.4 | 1.58 |
| **1** | 15.0 | 47.3 | 113.4 | 2.08 | 22.0 | 76.1 | 175.8 | 2.02 | 17.3 | 46.7 | 91.4 | 1.59 |
| **2** | 15.5 | 49.2 | 117.4 | 2.07 | 25.1 | 82.3 | 180.8 | 1.89 | 18.2 | 47.5 | 92.7 | 1.57 |
| **3** | 15.9 | 49.4 | 115.8 | 2.02 | 27.7 | 87.5 | 185.5 | 1.80 | 19.0 | 48.3 | 95.0 | 1.57 |
| **4** | 16.2 | 50.1 | 118.7 | 2.05 | 32.0 | 93.6 | 191.0 | 1.70 | 19.9 | 49.7 | 97.6 | 1.56 |
| **5** | 16.4 | 49.9 | 111.9 | 1.91 | 35.2 | 99.4 | 199.6 | 1.65 | 21.4 | 51.4 | 101.3 | 1.55 |
| **6** | 17.0 | 51.6 | 117.5 | 1.95 | 39.1 | 103.0 | 199.9 | 1.56 | 22.3 | 53.1 | 105.3 | 1.56 |
| **7** | 17.4 | 52.9 | 120.8 | 1.95 | 43.5 | 107.2 | 203.2 | 1.49 | 23.5 | 55.1 | 110.5 | 1.58 |
| **8** |  |  |  |  | 47.8 | 112.7 | 209.6 | 1.44 | 24.7 | 56.7 | 114.2 | 1.58 |
| **9** |  |  |  |  | 50.4 | 114.9 | 210.9 | 1.40 | 25.7 | 58.4 | 117.8 | 1.58 |
| **10** |  |  |  |  | 53.8 | 118.6 | 215.8 | 1.37 | 26.8 | 60.4 | 122.4 | 1.58 |

1. Sintratec Kit

All three formulations were successfully printed for 10 cycles on the Kit printer, as is illustrated in **Fig. S9**, which shows the tablet weight, tensile strength, volume, and density. Compared to data collected from SnowWhite^2^, interpretation is challenging due to larger variations in the data. Generally, the materials are discussed below in a similar fashion and the same order as for the SnowWhite^2^ printer, but in less depth, as the data collected for the samples printed with the Kit printer did not allow for the formation of suitable hypotheses. The data collected with the SnowWhite­^2^ printer provides a strong basis for hypothesis development, whereas the dataset obtained with the Kit lacks comparable strength and does not offer sufficient evidence to corroborate previous findings. The analytical data for the Kit printer is shown in **Section 3**. Not all the data is referenced or discussed, but is included for completeness.





**Fig. S9** *The average tablet weight (a), tensile strength (b), volume (c) and density (d) for the PVA, PVPVA and MAEA over the printing cycles printed in the Kit printer. The values shown are the mean ± s. The average tablet weights were determined with n = 32. The tensile strength was determined with the PTB 311E 800 (n = 5) while the volume and density were measured with calipers (n = 10).*

**3.1 PVA**

Ten cycles of the PVA formulation were successfully printed in the Kit printer. The printing series shows a significant (p<0.0001) difference in tablet weight between cycle 1 and cycle 2 (**Fig. S9**, orange). The initial tablet weight increase from 204.48 ± 9.67 cycle 1 to 216.4 ± 8.0 mg cycle 2 is also observed with the SnowWhite^2^ printing series (from 177.66 ± 5.1 mg cycle 1 to 204.2 ± 5.2 cycle 2) where the presence of moisture in the freshly prepared powder results in stickiness and reduced powder flow leading to lighter tablets. Print cycles 9 and 10 have a sudden increase in standard deviation and weight, which can be explained by slight layer shifting (**Fig. S10**). Layer shifting leads to more tablet weight and volume variation, as it is not consistent for all tablets. It also leads to increased weight as the laser can penetrate newly unprinted powder, whereas before the laser would penetrate the previously printed layers without melting additional powder. Layer shifting also occurred in the SnowWhite^2^ printer, but in a more severe form, where the tablets got dragged off the build platform in cycle 7. Overall, the PVA formulation is recyclable, and it is advisable to condition the material by preheating it once to get to the steady print state of the material.


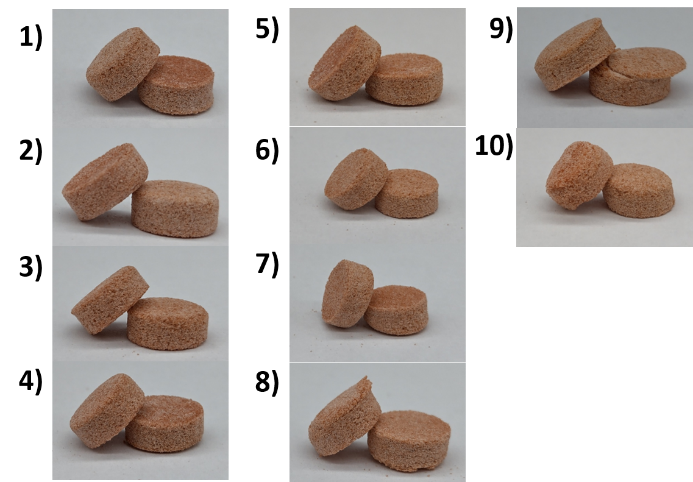


**Fig. S10** PVA based tablets cycle 1-10 printed with the Sintratec Kit printer, numbered corresponding to the cycle of printing.

**3.2 PVPVA**

The PVPVA formulation was also successfully cycled 10 times. **Fig. S9A** (Green) shows that the average tablet weight and tablet tensile strength decrease rapidly over the print cycles. The decrease in tablet weight is a result of a decrease in tablet volume in combination with a decrease in tablet density (**Fig. S9B**, Orange). The decrease in tablet density also leads to a decrease in tablet tensile strength. After cycle 5, the tablets were too weak for the breaking force to be consistently registered. The detection limit of the PTB 311E 800 was 10N, which corresponds to a tensile strength of 0.16 MPa based on the tablet dimensions. Therefore, the tensile strength of the tablets after Cycle 5 was approximately 0.16 MPa or lower. While this could be considered a print failure, the tablets could still be retrieved from the printer, so the experiment was continued. The PVPVA tablets produced by SnowWhite^2^ saw a similar but less strong decrease in tablet weight over time, paired with an increase in particle size of the printed material. For the Kit, the change in particle size is only minor (**Fig. S12B**). It is unclear what causes the rapid decrease in tablet mass, but the material is not recyclable under these conditions.

**3.3 MAEA**

Ten cycles were successfully printed with the 32-tablet design. **Fig. S9A, C, D** (Black) show that the tablet weight, volume, and density changes are seemingly random over the print cycles. The tensile strength (**Fig. S9B** (Black)) consistently decreases over the cycles. Under the used print conditions, this material behaves unpredictably and is not considered recyclable.

**3.4 Powder refreshment**

PVA and MAEA partially return to their starting tablet weight when 50% of fresh powder is added (**Fig. S11**), indicating that the formulation refreshment leads to remediation of the formulation printing properties to an earlier state. This is likely due to the reintroduction of moisture with the addition of the fresh powder and the corresponding increase in powder cohesion as explained in **section 3.1.1 and section 3.1.3** of the main text. PVPVA did not respond to formulation refreshment, and the tablet weight remained unchanged.





**Fig. S11** *The average tablet weight* *for the PVA, PVPVA, and MAEA over the printing cycles, including the 11^th^ cycle with 50% refreshed material printed with the SnowWhite^2^ printer.*

1. Analytical data Sintratec Kit

Particle size distribution

The particle size distributions of samples from all formulations printed using the Kit printer are shown below. The Dx10, Dx50, Dx90 and the span of the particle distribution are shown in **Table S3** for the 3 different formulations printed in the Sintratec Kit printer. The span is calculated with the equation S4.

**Table S3**

The Dx10, Dx50, Dx90 and the span of the particle size distributions obtained from the cycled formulations in the Sintratec Kit.

|  | **PVA** | | | | **PVPVA** | | | | **MAEA** | | | |
| --- | --- | --- | --- | --- | --- | --- | --- | --- | --- | --- | --- | --- |
| **Cycle**  **(#)** | **Dx10**  **(µm)** | **Dx50**  **(µm)** | **Dx90**  **(µm)** | **Span** | **Dx10**  **(µm)** | **Dx50**  **(µm)** | **Dx90**  **(µm)** | **Span** | **Dx10**  **(µm)** | **Dx50**  **(µm)** | **Dx90**  **(µm)** | **Span** |
| **Fresh** | 13.9 | 46.1 | 115.1 | 2.20 | 22.8 | 93.0 | 198.9 | 1.89 | 16.2 | 45.9 | 91.1 | 1.63 |
| **1** | 14.3 | 47.0 | 116.3 | 2.17 | 23.8 | 93.5 | 195.9 | 1.84 | 16.3 | 46.2 | 91.9 | 1.64 |
| **2** | 14.2 | 46.7 | 115.3 | 2.17 | 23.3 | 92.2 | 192.5 | 1.83 | 16.4 | 46.3 | 92.0 | 1.63 |
| **3** | 14.4 | 47.7 | 116.6 | 2.14 | 23.3 | 88.3 | 180.7 | 1.78 | 16.7 | 46.7 | 92.3 | 1.62 |
| **4** | 14.5 | 47.6 | 114.8 | 2.11 | 23.3 | 88.6 | 178.3 | 1.75 | 17.9 | 48.7 | 96.4 | 1.61 |
| **5** | 14.4 | 47.4 | 112.9 | 2.08 | 23.1 | 83.8 | 169.4 | 1.75 | 18.5 | 50.1 | 101.4 | 1.65 |
| **6** | 14.6 | 48.1 | 116.2 | 2.11 | 22.4 | 82.3 | 166.8 | 1.75 | 19.9 | 52.9 | 107.8 | 1.66 |
| **7** | 14.8 | 48.4 | 115.9 | 2.09 | 22.3 | 81.6 | 163.4 | 1.73 | 20.8 | 53.5 | 108.2 | 1.64 |
| **8** | 15.3 | 49.8 | 120.3 | 2.11 | 22.6 | 83.8 | 168.2 | 1.74 | 22.2 | 55.3 | 111.2 | 1.61 |
| **9** | 14.9 | 49.2 | 118.6 | 2.11 | 22.6 | 83.7 | 167.5 | 1.73 | 22.0 | 54.4 | 109.1 | 1.60 |
| **10** | 15.3 | 50.6 | 121.0 | 2.09 | 22.3 | 84.6 | 170.5 | 1.75 | 21.2 | 54.2 | 109.8 | 1.63 |


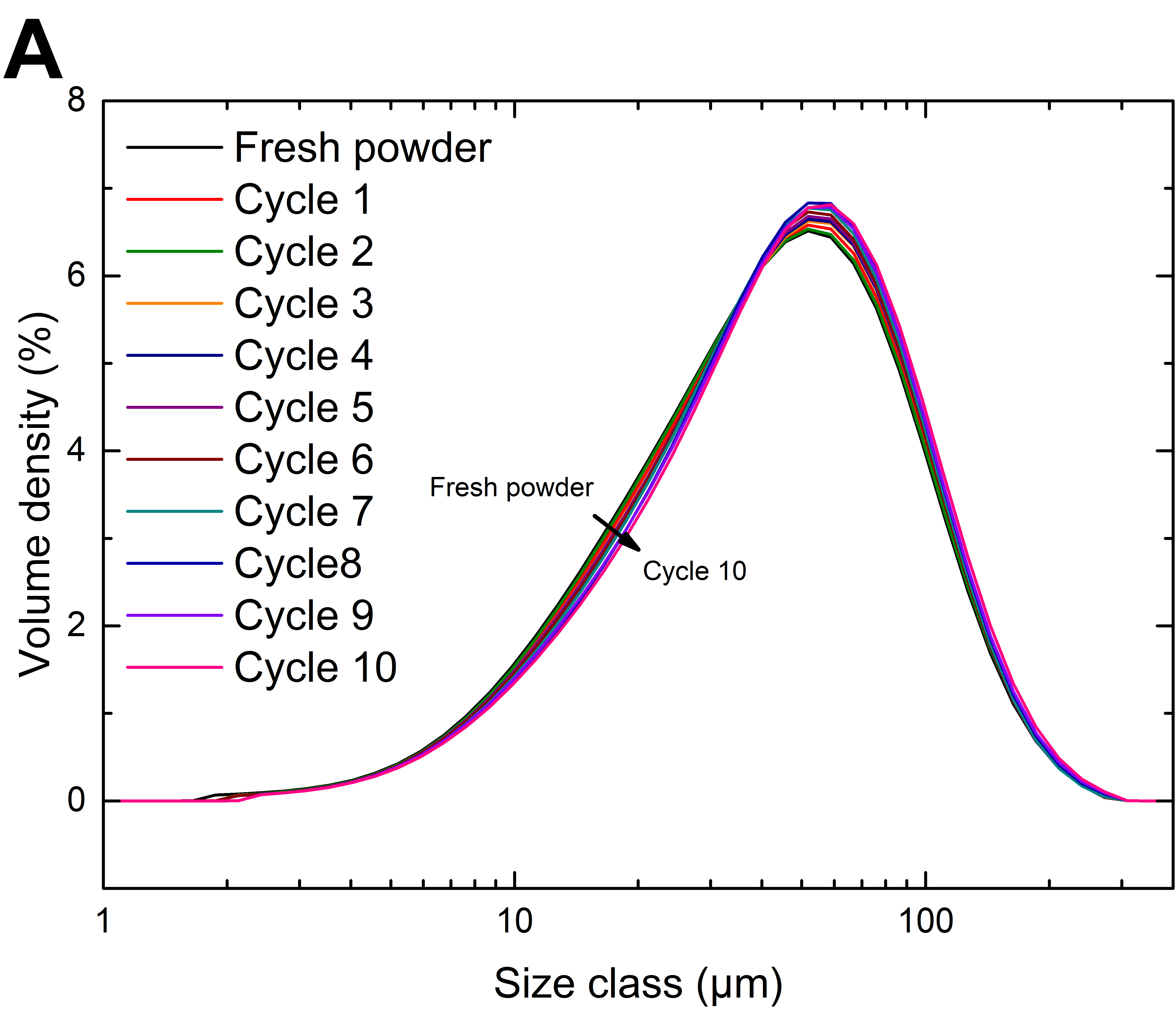


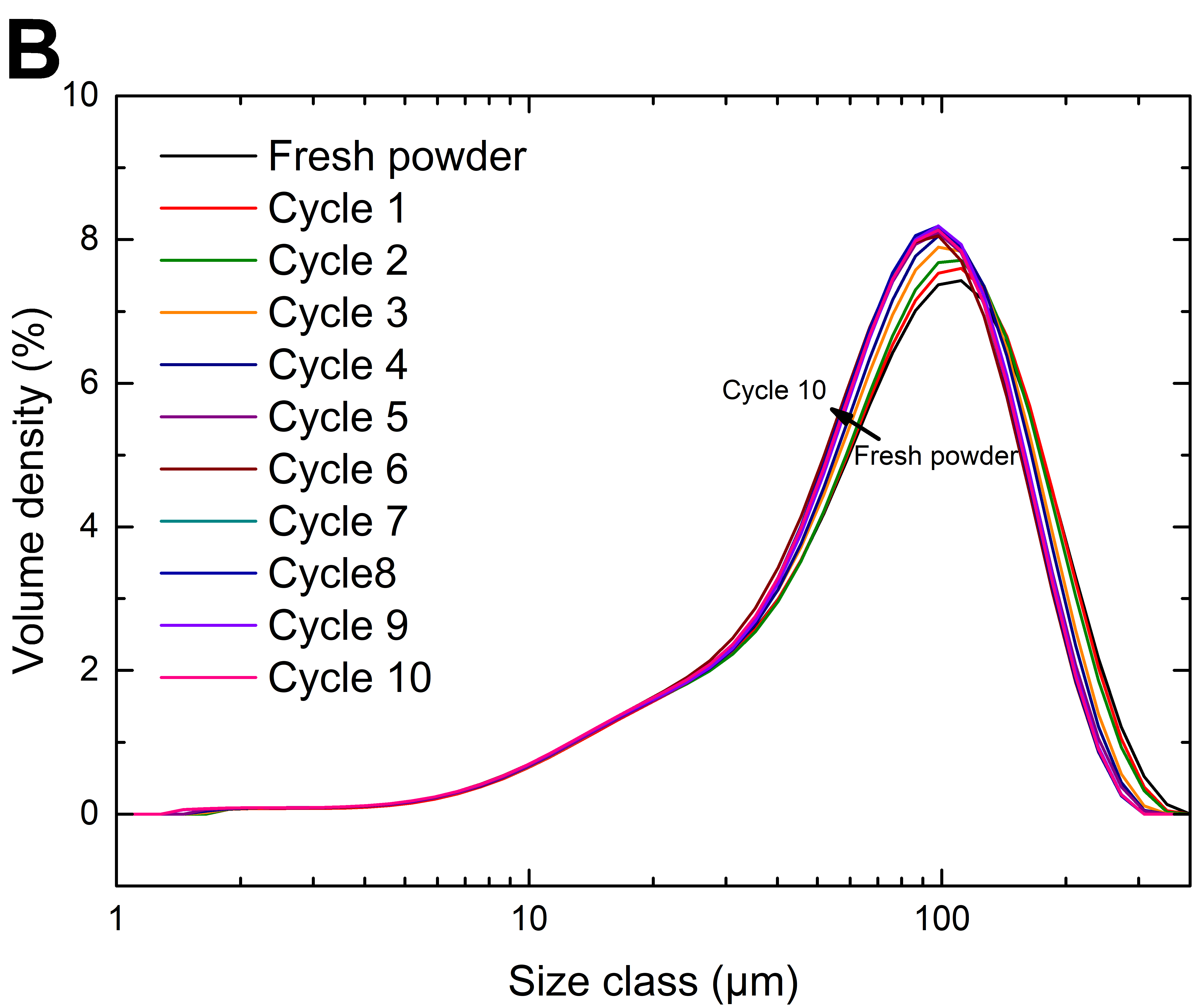

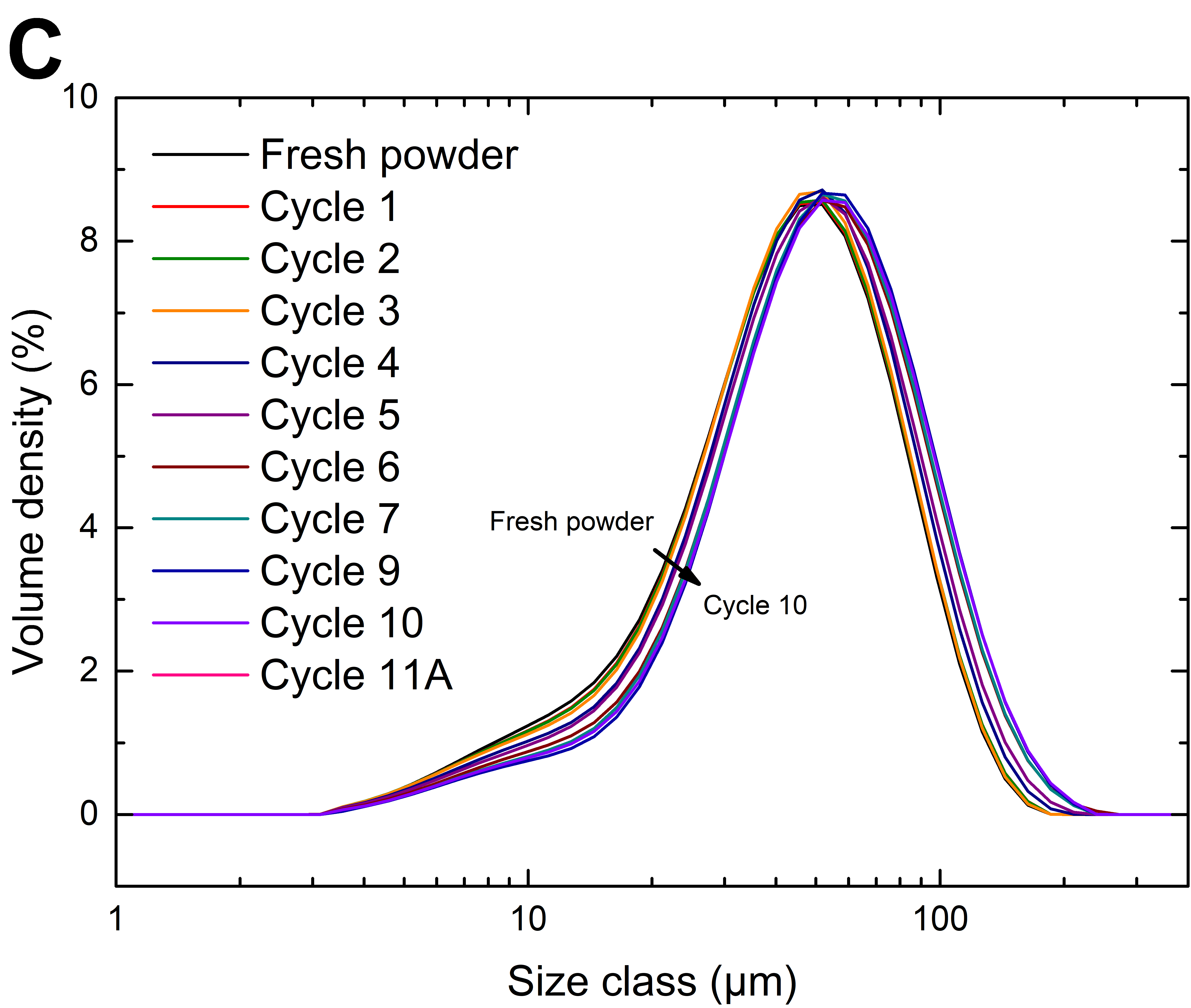


**Fig. S12** Particle size distribution of the PVA (A), PVPVA (B), and MAEA (C) formulations taken over the ageing cycles performed in the Kit printer. The arrows indicate the changes in particle size. Note, the overall particle size of the PVPVA formulation gets smaller.

Differential scanning calorimetry (DSC)

The DSC thermograms from all samples obtained with the Kit are shown below. The Glass transition temperatures for all 3 formulations printed on the Kit are shown in **Table S1**.

**Table S4**

The Glass transition temperatures for all 3 formulations printed on the Sintratec kit printer.

|  | Sintratec Kit | | |
| --- | --- | --- | --- |
| Cycle | PVA | PVPVA | MAEA |
|  | T_g_ (°C) | T_g_ (°C) | T_g_ (°C) |
| 1 | 59.5 | 106.4 | 121.4 |
| 2 | 61.4 | 105.6 | 114.9 |
| 3 | 60.4 | 106.4 | 115.2 |
| 4 | 61.1 | 108.1 | 112.4 |
| 5 | 60.4 | 105.9 | 114.2 |
| 6 | 60.3 | 106.3 | 114.0 |
| 7 | 60.7 | 108.8 | 115.2 |
| 8 | 61.9 | 104.5 | 113.4 |
| 9 | 61.6 | 107.1 | 112.7 |
| 10 | 61.7 | 104.4 | 111.3 |

**
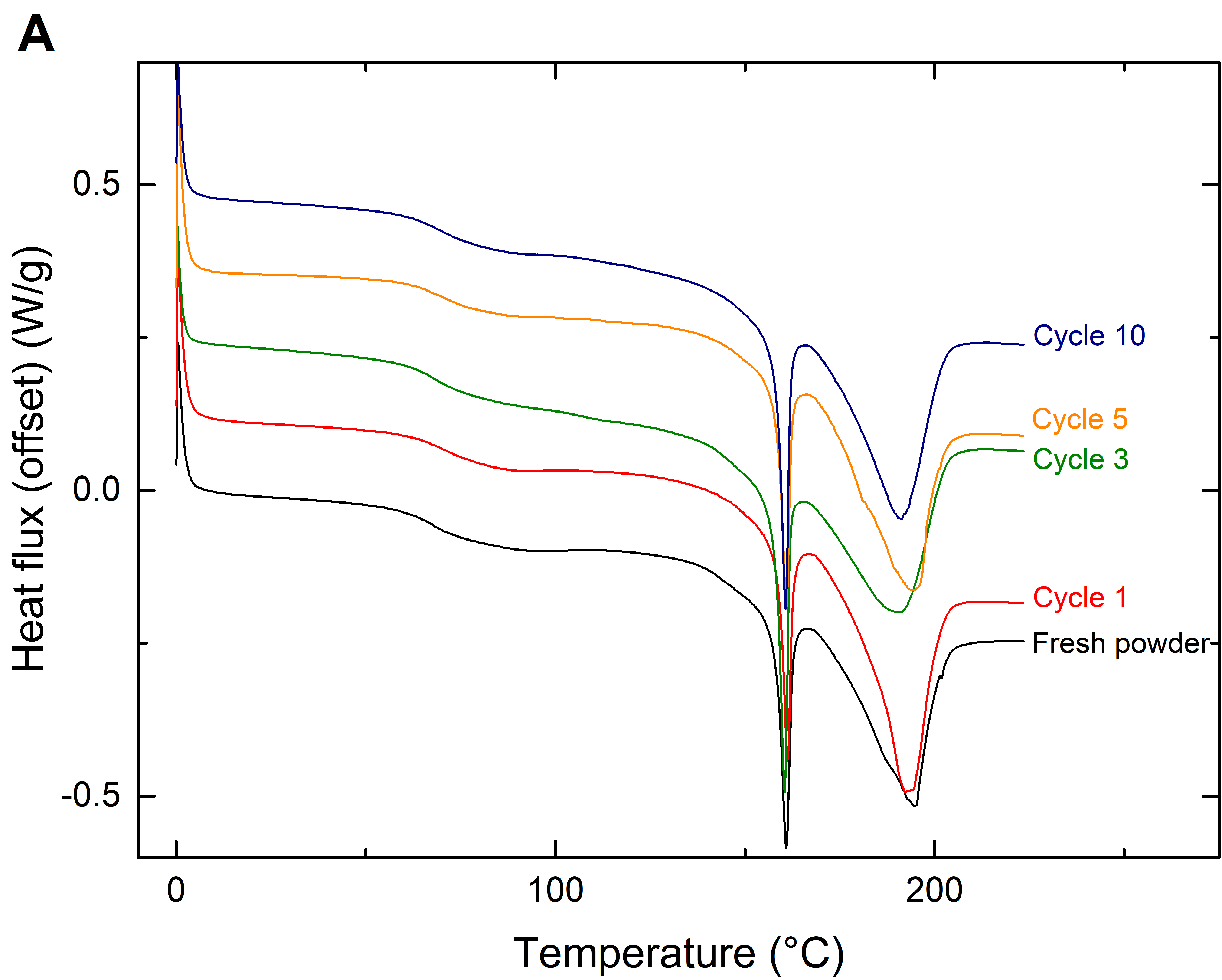
**

**
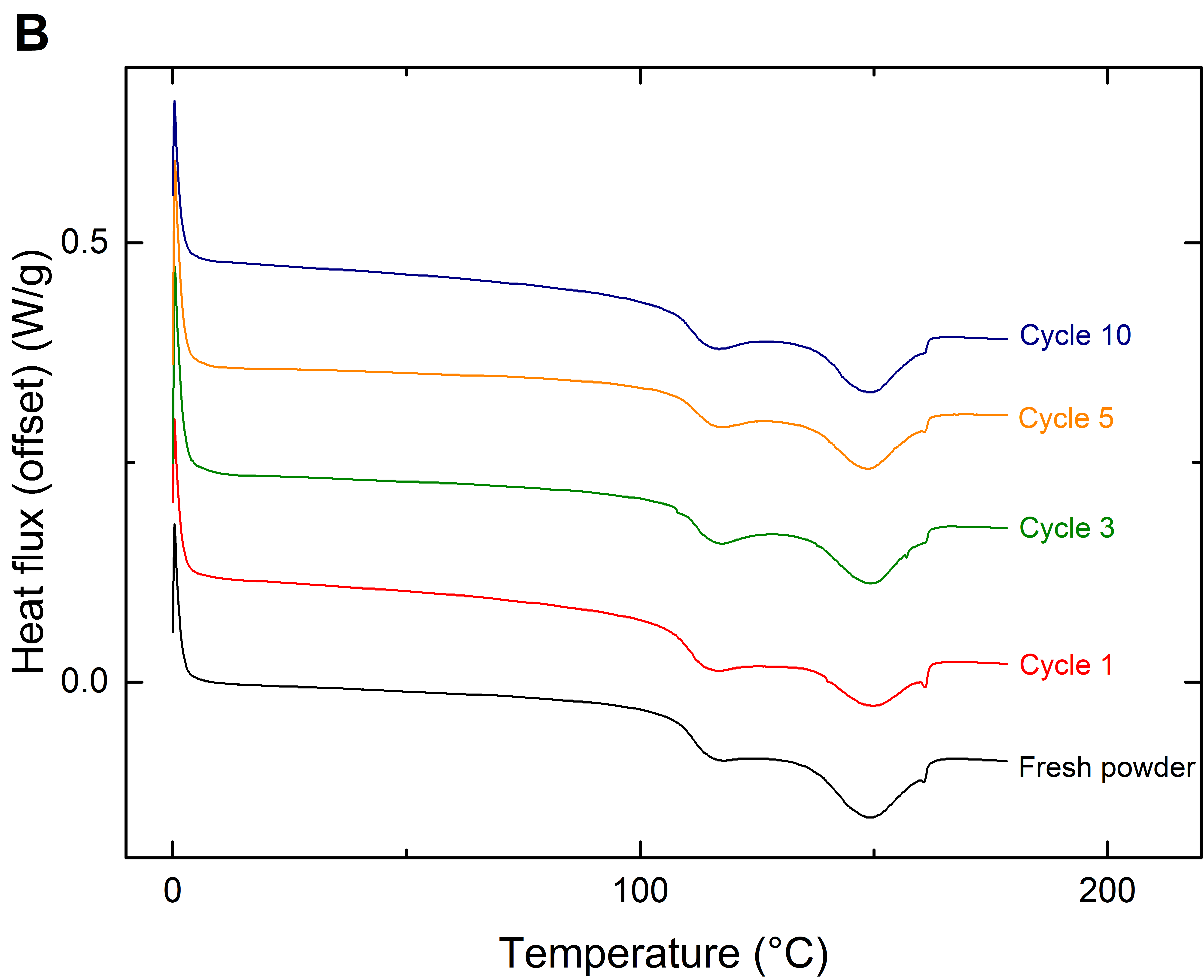

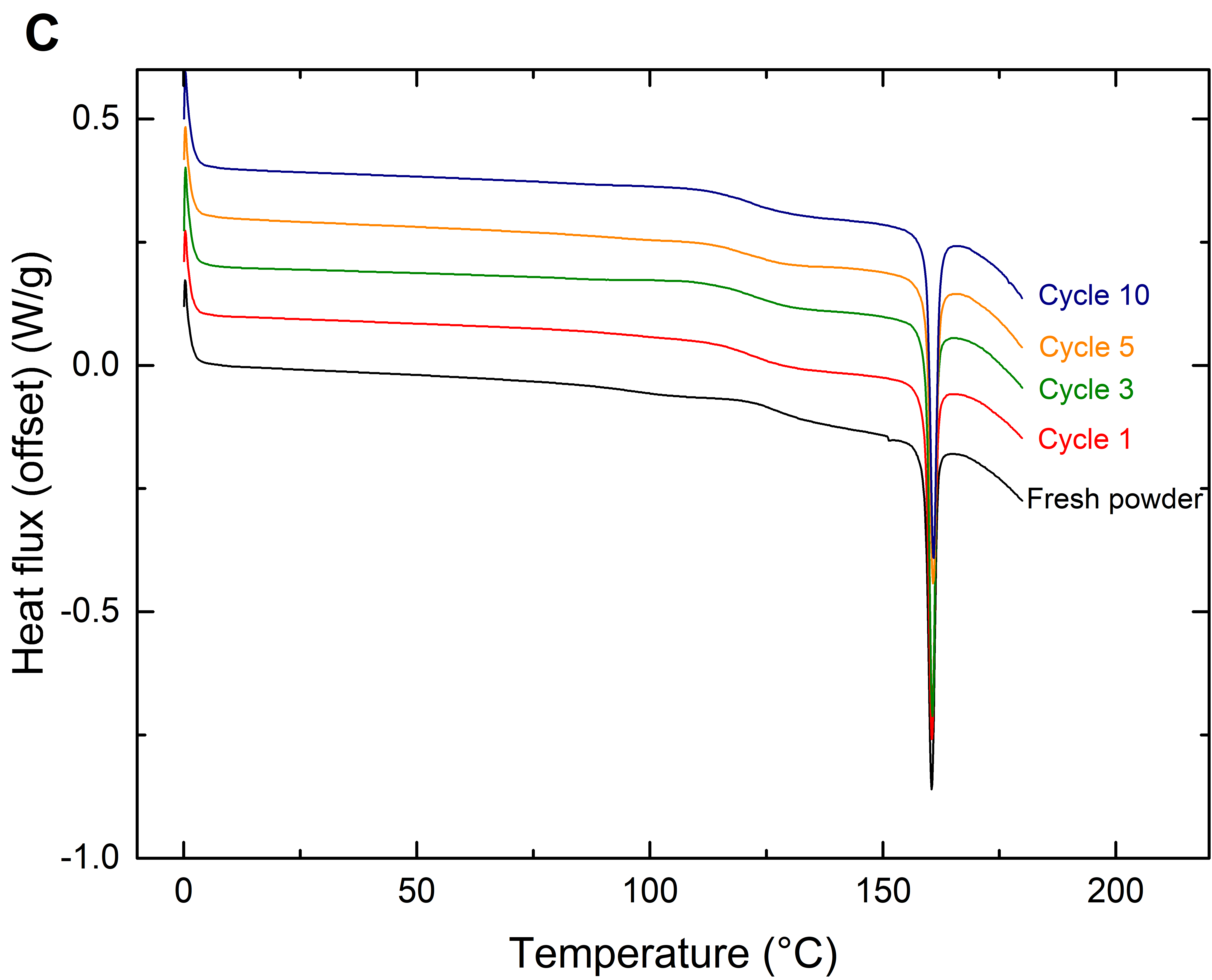
**

**Fig. S13** *DSC thermographs of the PVA (A), PVPVA (B), and MAEA (C) formulations measured over the ageing cycles performed in the Kit printer. Samples were measured in the absence of moisture.*

Thermogravimetric analysis (TGA)

The TGA graphs of samples from all formulations printed using the Kit printer are shown below.

**
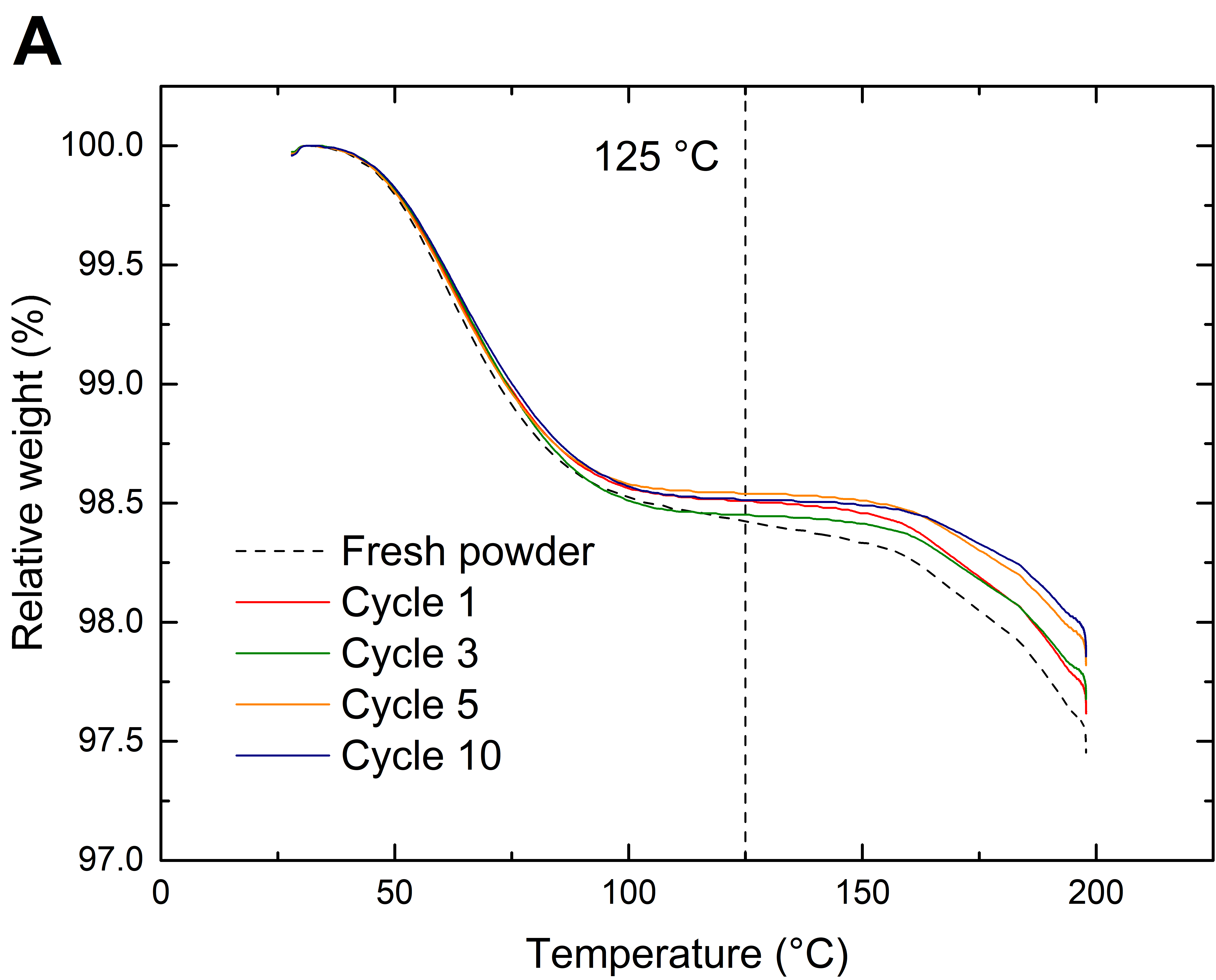
**

**
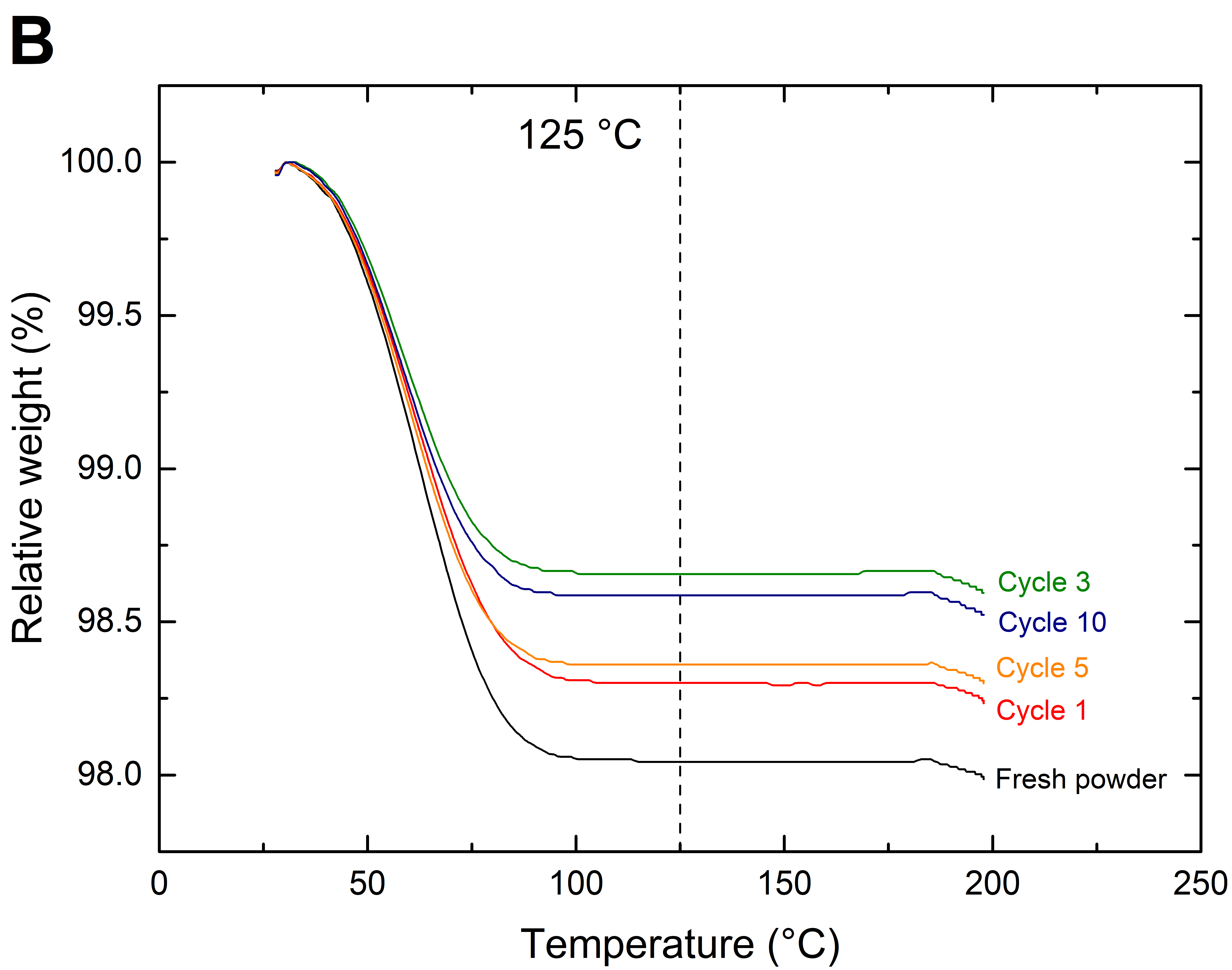
**

**
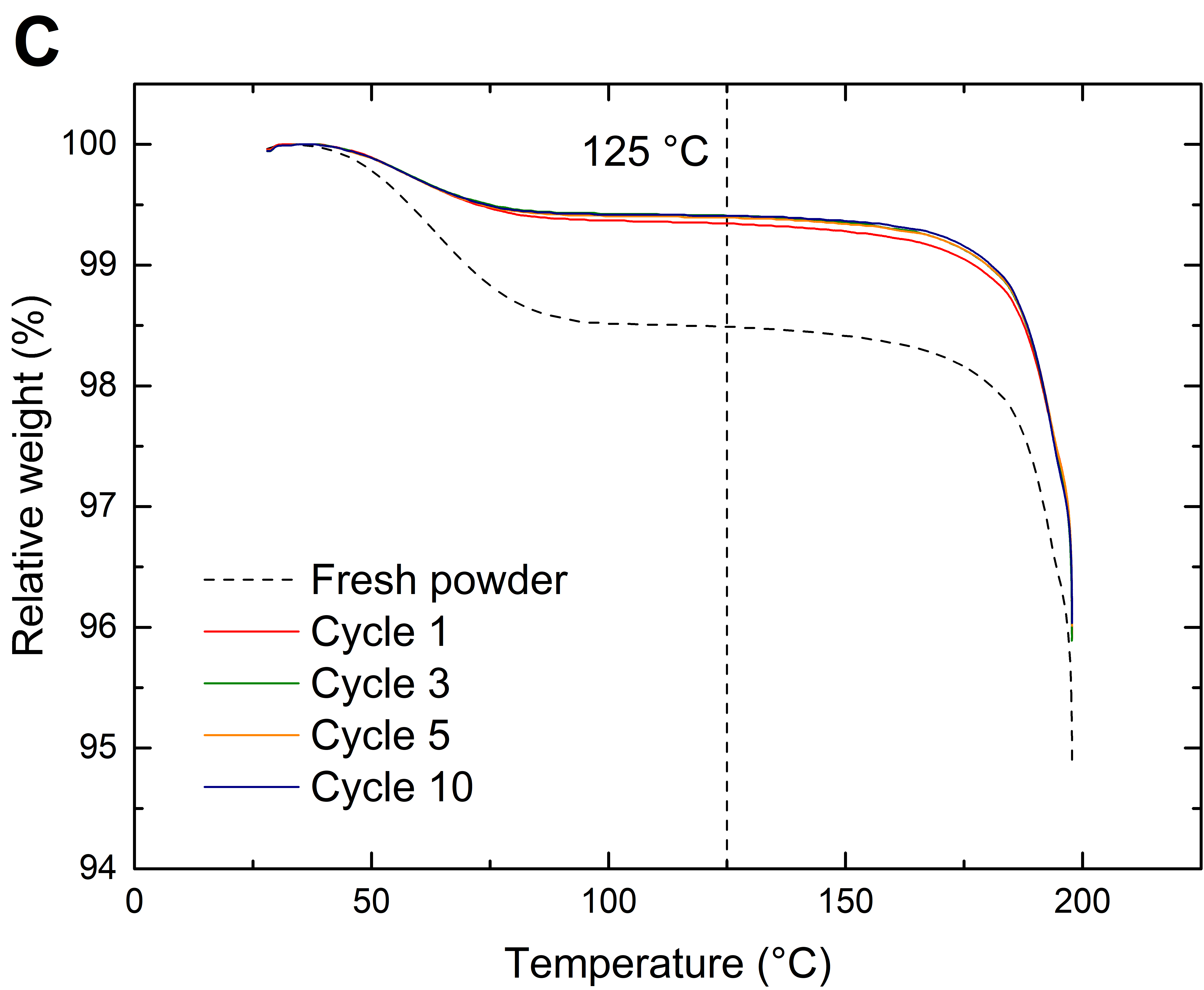
**

**Fig. S14** *TGA thermographs of the PVA (A), PVPVA (B), and MAEA (C) formulation taken over the ageing cycles performed in the Kit printer. The dashed line indicates the temperature at which the relative weight loss was determined as loss on drying. Measurements were performed on the TGA/DSC 3+.*

**X-ray powder diffraction** (XRPD)

The X-ray powder diffractograms of samples from all formulations printed using the Kit printer are shown below.










**Fig. S15** X-ray powder diffractograms of the PVA (A), PVPVA (B), and MAEA (C) formulations taken over the ageing cycles performed in the Kit printer. These diffractograms were measured using Cu Kα radiation.

Fourier transformed infra-red spectroscopy (FTIR)

The FTIR spectra of samples from all formulations printed using the Kit printer are shown below.

**
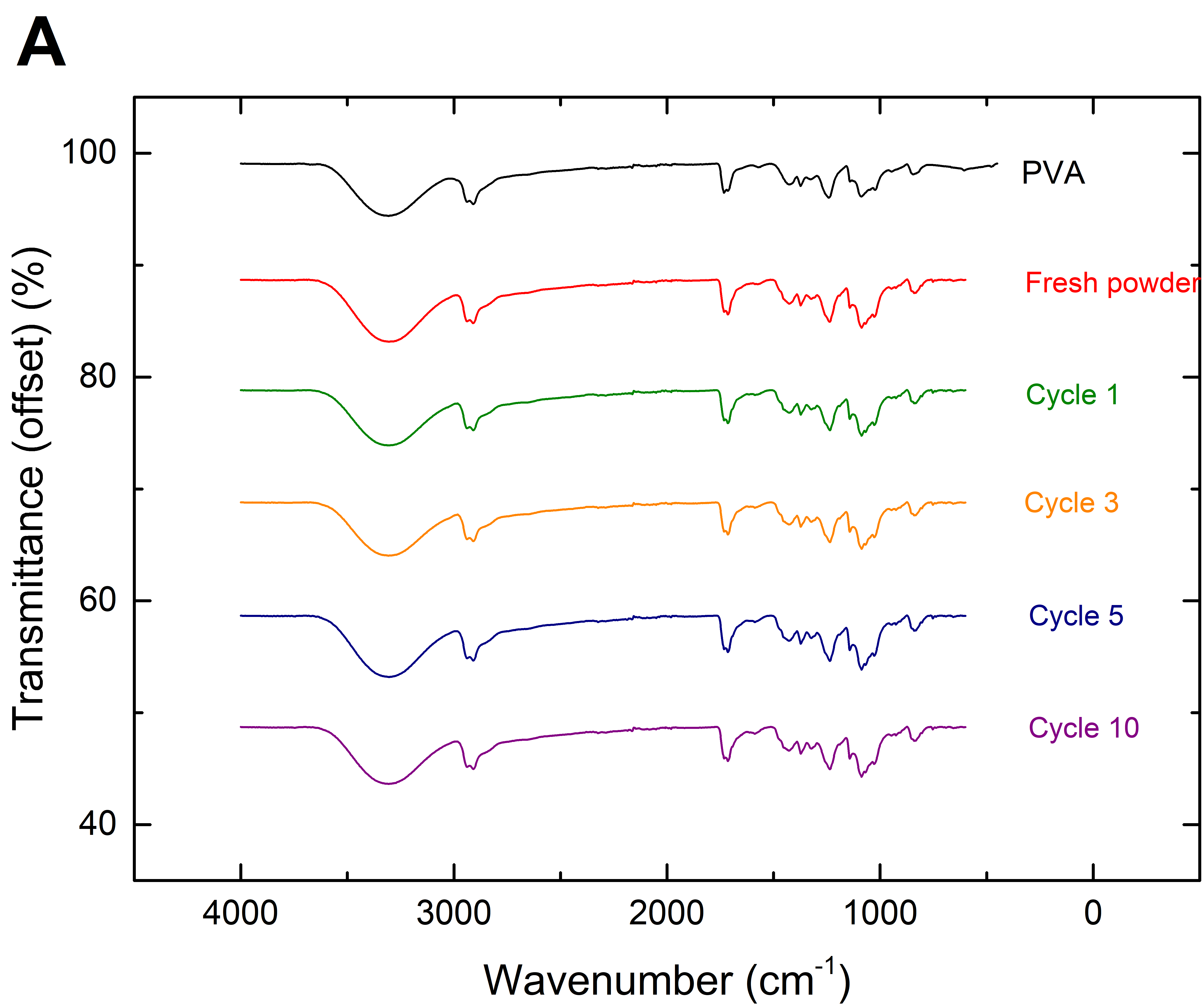
**

**
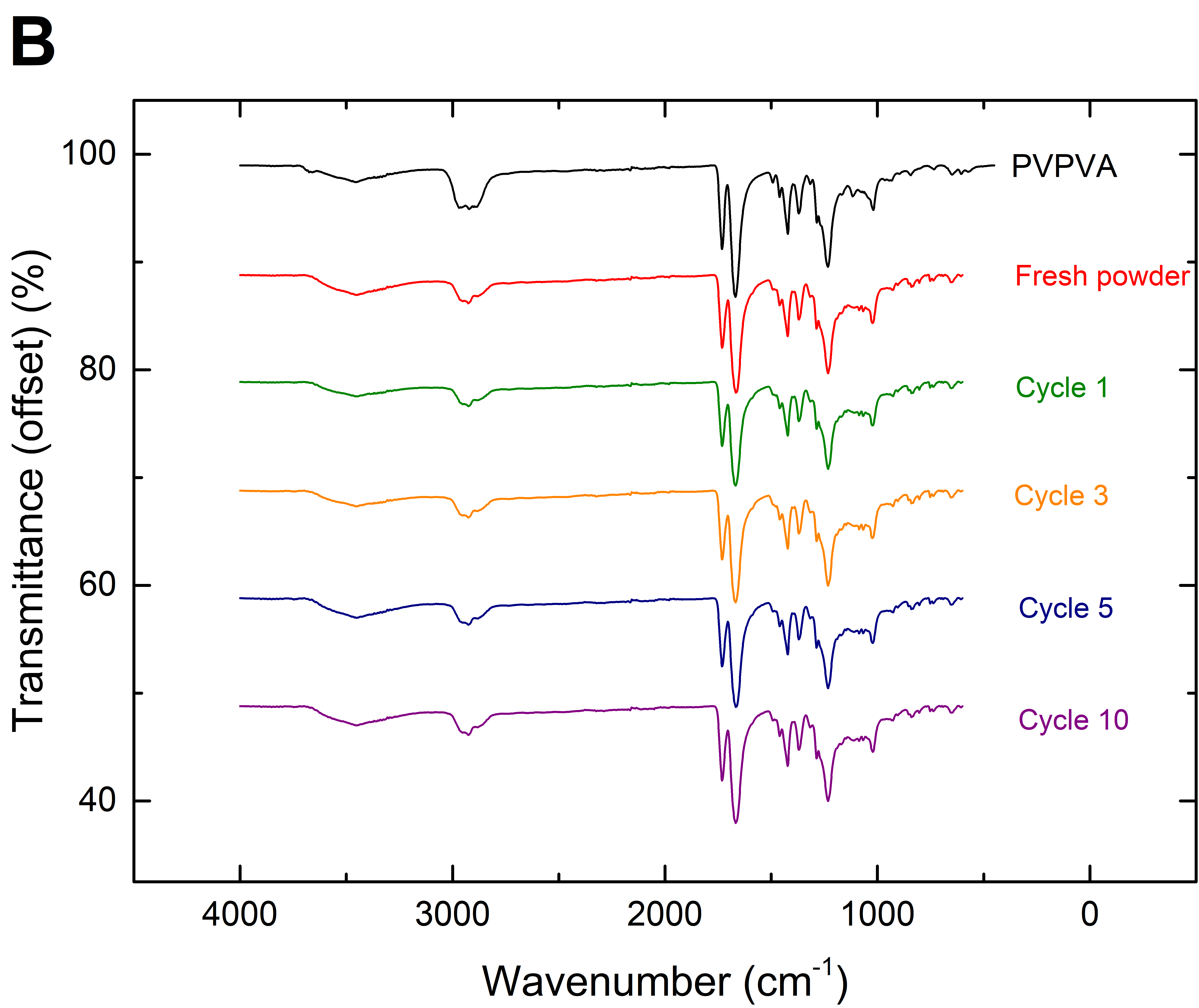
**


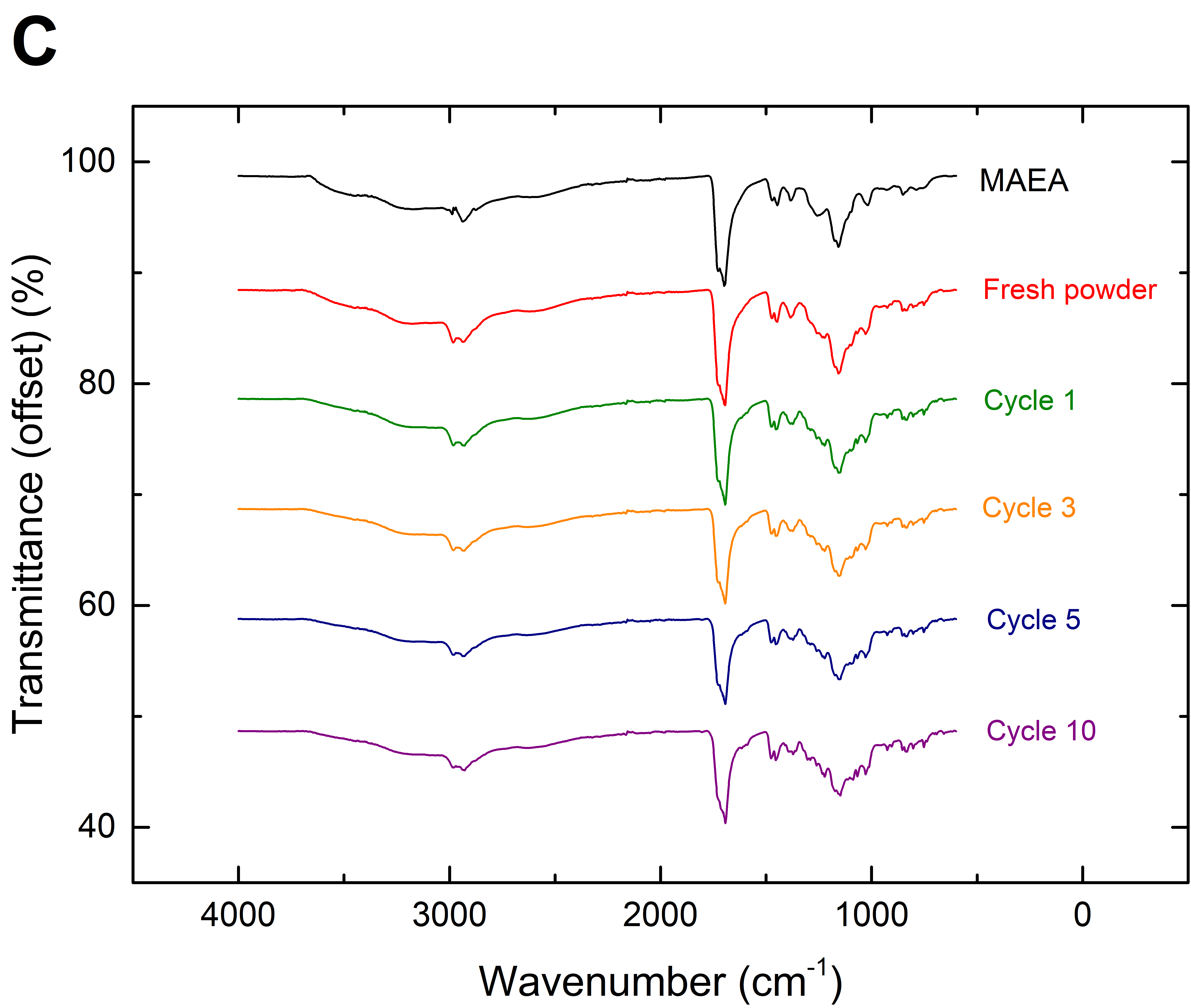


**Fig. S16** FTIR spectra of the PVA (A), PVPVA (B), and MAEA (C) formulations taken over the ageing cycles performed in the Kit printer.

Powder rheology

The granudrum data of samples from all materials printed using the Kit printers are shown below.


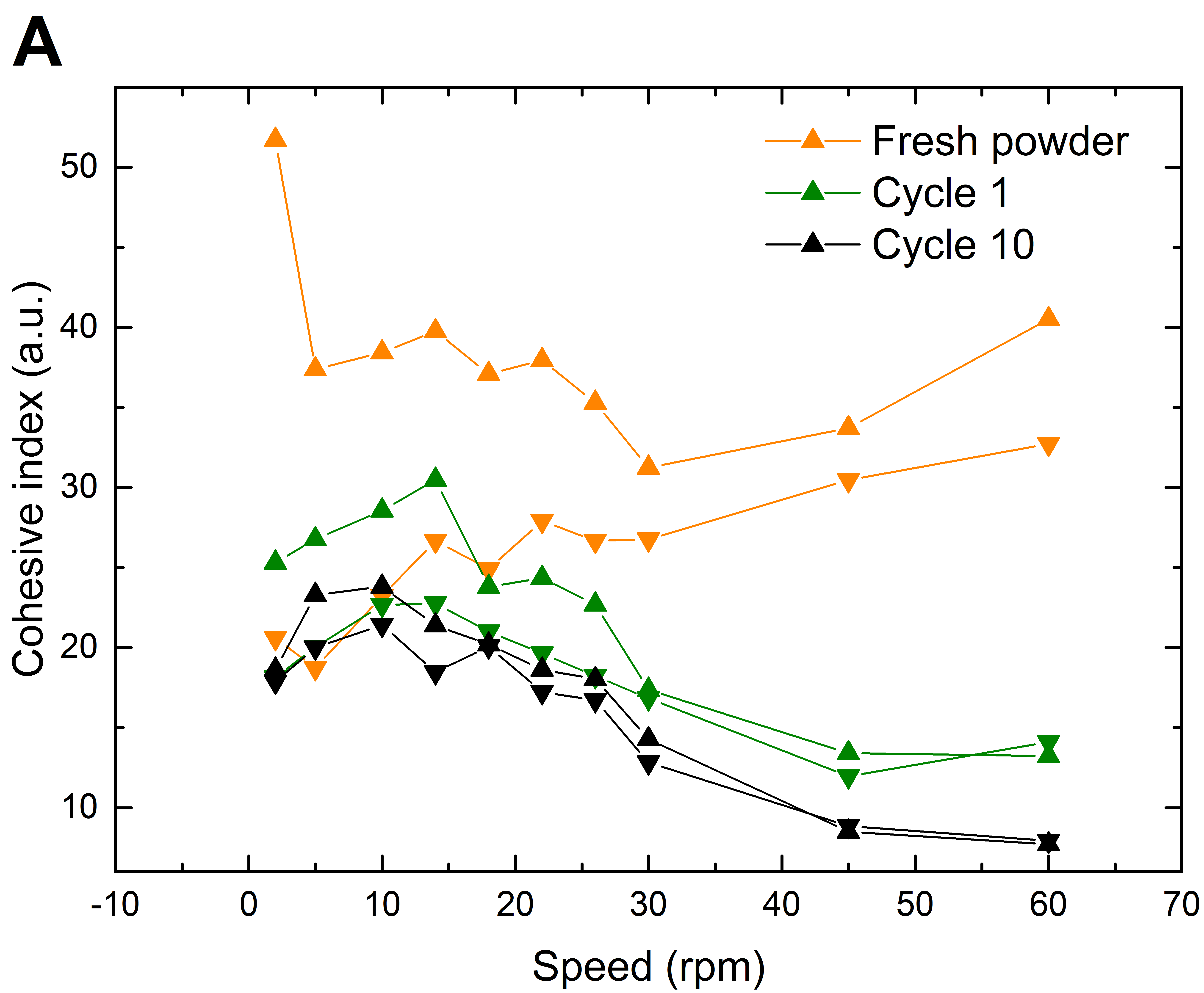


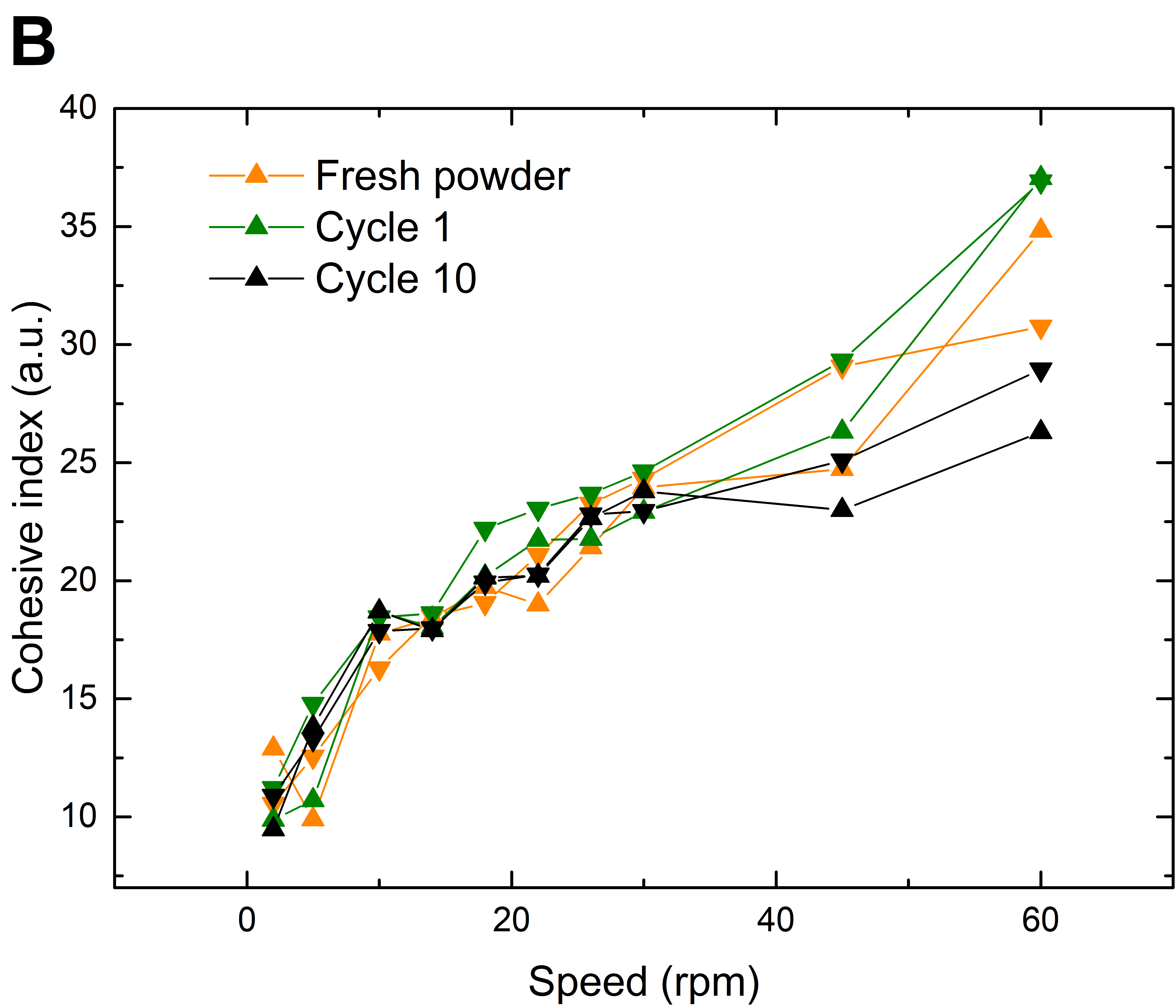


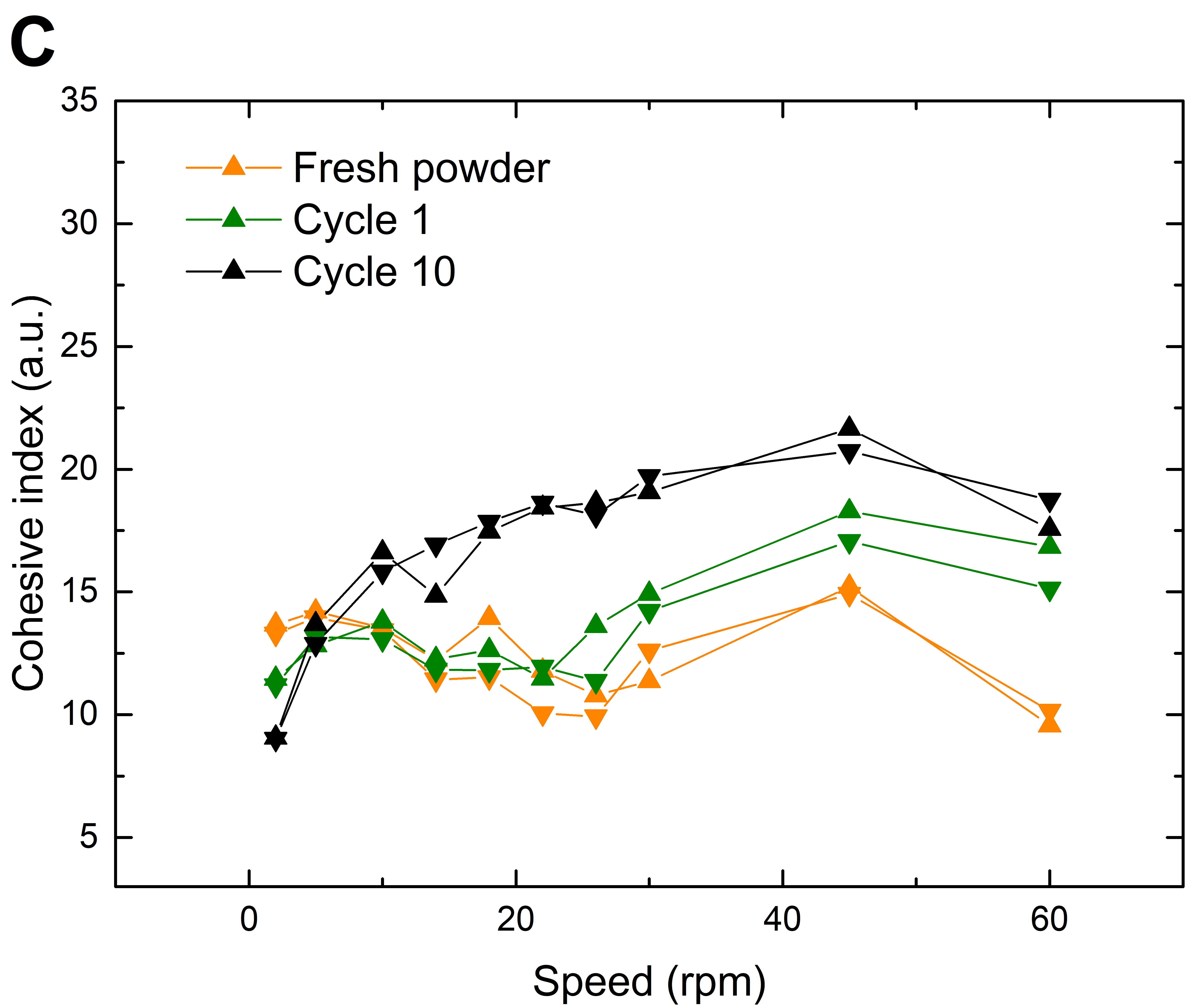


**Fig. S17** Cohesive index at different rotational speeds for the PVA (A), PVPVA (B), and MAEA (C) based formulations taken over the ageing cycles performed in the Kit printer. The triangles pointing up indicate the acceleration sequence while the triangles pointing down indicate the deceleration sequence.

1. Equations

Herring’s scaling law:

$$\begin{aligned} \frac{\Delta t_{a}}{\Delta t_{b}}=\left( \frac{R_{a,0}}{R_{b,0}} \right)^{m} \#\left( S SEQ EQUATION 2 \right) \end{aligned}$$

where subscript a and b denote two different powders of initial particle sizes R_a,0_ and R_b,0,_ respectively (Herring, 1950). Δt_a_ and Δt_b_ represent the time the different powder needs to reach an equivalent degree of sintering. The integer *m* corresponds to a value between 1 and 4 depending on the mass transport mechanism (Kang, 2005; Song H.and Coble, 1984).

Gordon and Taylor equation:

$$\begin{aligned} T_{g,m}= \frac{X_{w}T_{g,w}+ {kX}_{s}T_{g,s}}{X_{w}+ {kX}_{s}}\#\left( S SEQ EQUATION 3 \right) \end{aligned}$$

Where subscript m, w and s denote the mixture, water, and the solid state, respectively (Gordon and Taylor, 1952). The T_g_ is the glass transition temperature, X is the mass fraction, and k is the Gordon-Taylor parameter (Sablani et al., 2007).

Williams-Landel-Ferry (WLF) equation:

$$\begin{aligned} log\left( \frac{\eta_{T}}{\eta_{T_{g}}} \right)= \frac{-C\left( T- T_{g} \right)}{B+\left( T- T_{g} \right)}\#\left( S SEQ EQUATION 4 \right) \end{aligned}$$

where T and T_g_ denote the temperature and glass transition temperature (Williams et al., 1955). η is the viscosity. B and C are fitting parameters with values of -17.4 and 51.6 K respectively suitable for most polymers as investigated by *Williams et al* (Williams et al., 1955)*.*

References

Gordon, M., Taylor, J.S., 1952. Ideal copolymers and the second‐order transitions of synthetic rubbers. i. non‐crystalline copolymers. Journal of Applied Chemistry 2, 493–500. https://doi.org/10.1002/jctb.5010020901

Herring, C., 1950. Effect of Change of Scale on Sintering Phenomena. J Appl Phys 21, 301–303. https://doi.org/10.1063/1.1699658

Kang, S.-J., 2005. Sintering, densification, grain growth, and microstructure., Elsevier Butterworth-Heinemann.

Sablani, S.S., Kasapis, S., Rahman, M.S., 2007. Evaluating water activity and glass transition concepts for food stability. J Food Eng 78, 266–271. https://doi.org/10.1016/j.jfoodeng.2005.09.025

Song H. and Coble, R.L. and B.R.J., 1984. The Applicability of Herring’s Scaling Law to the Sintering of Powders, in: Kuczynski George Czeslaw and Miller, A.E. and S.G.A. (Ed.), Materials Science Research: Volume 16 Sintering and Heterogeneous Catalysis. Springer US, Boston, MA, pp. 63–79. https://doi.org/10.1007/978-1-4613-2761-5_5

Williams, M.L., Landel, R.F., Ferry, J.D., 1955. The Temperature Dependence of Relaxation Mechanisms in Amorphous Polymers and Other Glass-forming Liquids. J Am Chem Soc 77, 3701–3707. https://doi.org/10.1021/ja01619a008
